# Hypervalent Iodine-Mediated Diastereoselective α-Acetoxylation of Cyclic Ketones

**DOI:** 10.3389/fchem.2020.00467

**Published:** 2020-07-10

**Authors:** Jiashen Tan, Weiqin Zhu, Weiping Xu, Yaru Jing, Zhuofeng Ke, Yan Liu, Keiji Maruoka

**Affiliations:** ^1^School of Chemical Engineering and Light Industry, Guangdong University of Technology, Guangzhou, China; ^2^PCFM Lab, School of Materials Science and Engineering, Sun Yat-sen University, Guangzhou, China; ^3^Graduate School of Pharmaceutical Sciences, Kyoto University, Kyoto, Japan

**Keywords:** α-acetoxylation, hypervalent iodine (III) reagent, cyclic ketones, diastereoselectivity, density functional theory

## Abstract

A binary hybrid system comprising a hypervalent iodine(III) reagent and BF_3_•OEt_2_ Lewis acid was found to be effective for the diastereoselective α-acetoxylation of cyclic ketones. In this hybrid system, BF_3_•OEt_2_ Lewis acid allowed the activation of the hypervalent iodine(III) reagent and cyclic ketones for smooth α-acetoxylation reaction, achieving high diastereoselectivity. This hypervalent iodine-mediated α-acetoxylation of the cyclic ketone reaction plausibly undergoes an S_N_2 substitution mechanism via an α-*C*-bound hypervalent iodine intermediate. The diastereoselectivity of the reaction mainly originates from thermodynamic control.

## Introduction

**Scheme 1:**
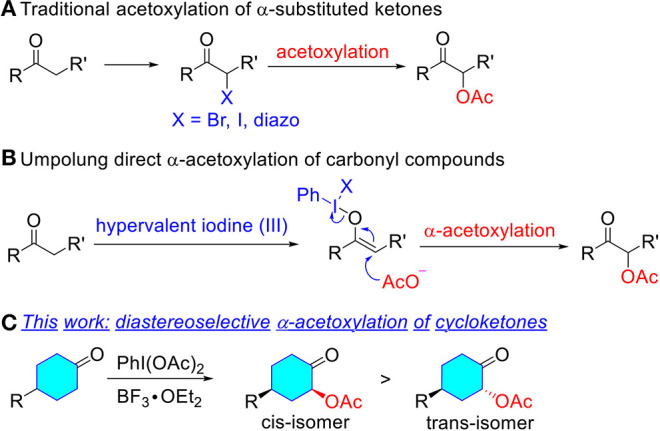
Different methods for the α-acetoxylation reaction of ketones. **(A)** Traditional acetoxylation of α-substituted ketones. **(B)** Umpolung direct α-acetoxylation of carbonyl compounds. **(C)** This work: diastereoselective α-acetoxylation of cycloketones.

The direct α-acetoxylation of ketones provides an efficient and synthetically practical method for α-oxygen functionalization of carbonyl compounds, which exhibits various applications as synthetic key precursors and versatile synthons in organic synthesis (Mizukami et al., 1978; Hecker and Werner, 1993; Varma et al., 1998; Kaila et al., 2007; Edwards et al., 2008; Richter et al., 2018; Huang et al., 2019). Therefore, considerable efforts have been made in the development of synthetic protocols for α-acetoxylation of ketones (Scheme 1). Traditional methods to prepare α-acetoxy ketones are the acetolysis of α-diazo ketone (Newman and Beal, 1950; Erickson et al., 1951; Corey and Knapp, 1976; Kitamura et al., 2012; Wang et al., 2014; Tan et al., 2016; Yuan et al., 2016; Zhang et al., 2016; Hu et al., 2018), α-bromo ketone (Tanner et al., 1991; Valgimigli et al., 2003; Ahmed and Langer, 2006; Chen et al., 2013, 2016; Nolla-Saltiel et al., 2014; Carneiro et al., 2015; Liu et al., 2016; Yuan et al., 2019), and *in situ* generated α-iodo derivatives (Du et al., 2015; Ren et al., 2016; Chen et al., 2017; Tan et al., 2017; Pogaku et al., 2019), which usually require the pre-functionalization of ketones (Scheme 1A). In contrast, umpolung reactions have taken a prominent place for the direct α-oxygenation of carbonyl compounds via oxidation of the enolates or related derivatives with transition metal complexes (Littler, 1962; Heiba and Dessau, 1971; Ng and Henry, 1976; Rubottom et al., 1983; El-Qisairi and Qaseer, 2002; Hamed et al., 2012), or hypervalent iodine reagents (Mizukami et al., 1978; Nicolaou et al., 2002; Bogevig et al., 2004; Sunden et al., 2004; Ochiai et al., 2005; Huang et al., 2007; Yu et al., 2010; Izquierdo et al., 2016; Arava et al., 2017) (Scheme 1B). Metal-free hypervalent iodine compounds as classical oxidative functionalization reagents have attracted considerable interest due to a variety of advantages relative to conventional oxidants such as heavy metals (Pb, Tl, or Hg), including low toxicity, ready availability, mild conditions, excellent selectivity, and a comparable reactivity (Du et al., 2015; Ren et al., 2016; Chen et al., 2017). However, efficient and practical methods for the diastereoselective α-acetoxylation of cyclic ketones have not yet been well-developed.

As one of our ongoing interests (Sakamoto et al., 2016, 2017, 2018; Liu et al., 2017; Selvakumar et al., 2017; Shu et al., 2019) to construct selective methods for α-functionalization of carbonyl compounds, we have become keenly interested in the possibility of the diastereoselective α-acetoxylation of cyclic ketones (Scheme 1C). In this context, we wish to report our initial study on creating a hybrid system comprised of hypervalent iodine(III) reagent and BF_3_•OEt_2_.

## Results and Discussion

We began our study on the diastereoselective α-acetoxylation reaction of cyclic ketone with hypervalent iodine(III) reagent and certain acid as an activator. As shown in Table 1, the reaction of 4-phenylcyclohexanone with (diacetoxyiodo)benzene in acetic acid solvent at room temperature for 24 h resulted in almost recovery of the starting ketone (Table 1, entry 1). In the presence of additives such as CF_3_CO_2_H, α-acetoxylation product **1** was obtained in low yields (Table 1, entry 2), whereas the use of Lewis acids ZnCl_2_ and AlCl_3_ as additives resulted in no target product even though 4-phenylcyclohexanone was fully consumed under the applied reaction conditions (Table 1, entries 5 and 6). Fortunately, addition of Brønsted acid TfOH (Table 1, entry 3), and Lewis acids AgOTf, Sc(OTf)_3_, Mg(OTf)_2_, Cu(OTf)_2_, Zn(OTf)_2_, and BF_3_•OEt_2_ (Table 1, entries 4, 7–11) improved the yield of the product **1**. BF_3_•OEt_2_ gave rise to desired *cis*-isomer of α-acetoxylation product, *cis*-**1**, in good yield (Table 1, entry 11). Increasing the loading of BF_3_•OEt_2_ from 1 equiv to 3 equiv resulted in an increased selectivity (Table 1, entry 13). However, reducing the reaction time from 24 h to 12 or 6 or 2 h resulted in a gradual decrease in selectivity toward *cis*-**1** (Table 1, entries 15–17). Further increasing the BF_3_•OEt_2_ loading to 5 equiv, or extending the reaction time (48 h), or enhancing the reaction temperature to 50°C, however, did not increase the transformation or selectivity (Table 1, entries 14, 18, and 19). Lewis acid BF_3_•OEt_2_ plays a key role in the α-acetoxylation of cyclohexanone, which should be attributed to the enolization of cyclohexanone to enol that is essential for the reaction to smoothly take place (Ochiai et al., 2005). Changing acetic acid solvent to other organic solvents such as CH_2_Cl_2_, EtOAc, Et_2_O, toluene, CH_3_CN, CF_3_CH_2_OH, and (CF_3_)_2_CHOH even in the presence of 10 equiv acetic acid resulted in the decrease of both yield and selectivity (Table 1, entries 20–32). Therefore, the optimal condition for the synthesis of *cis*-**1** was established as follows: Reaction of 4-phenylcyclohexanone (1 equiv) with PhI(OAc)_2_ (1.5 equiv) and BF_3_•OEt_2_ (3 equiv) in acetic acid solvent at room temperature for 24 h. The α-acetoxylation of 4-phenylcyclohexanone proceeded smoothly in ten-gram scale under this optimal reaction condition, without a lose of reactivity and selectivity. The configuration of the major α-acetoxylation product was determined as *cis*-isomer by a single-crystal X-ray diffraction analysis (Table 1).

**Table 1:** Diastereoselective α-acetoxylation of ketone with hypervalent iodine(III) reagents in the presence of additives^a^.

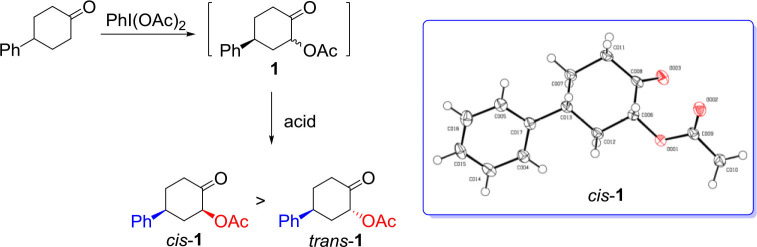				
**Entry**	**Solvent**	**Additive (equiv)**	**Condition (°C, h)**	**% yield of 1^b^ (*cis*/*trans* ratio)**
1	AcOH	—	RT, 24	Trace
2	AcOH	CF_3_CO_2_H (1)	RT, 24	17 (1.4:1)
3	AcOH	TfOH (1)	RT, 24	54 (12.2:1)
4	AcOH	AgOTf (1)	RT, 24	37 (2.1:1)
5	AcOH	ZnCl_2_ (1)	RT, 24	0
6	AcOH	AlCl_3_ (1)	RT, 24	0
7	AcOH	Sc(OTf)_3_ (1)	RT, 24	42 (7.0:1)
8	AcOH	Mg(OTf)_2_ (1)	RT, 24	52 (2.1:1)
9	AcOH	Cu(OTf)_2_ (1)	RT, 24	60 (3.7:1)
10	AcOH	Zn(OTf)_2_ (1)	RT, 24	68 (3.0:1)
11	AcOH	BF_3_•OEt_2_ (1)	RT, 24	70 (6.8:1)
12	AcOH	BF_3_•OEt_2_ (2)	RT, 24	73 (10.5:1)
13	AcOH	BF_3_•OEt_2_ (3)	RT, 24	67 (11.8:1)
14	AcOH	BF_3_•OEt_2_ (5)	RT, 24	71 (12.5:1)
15	AcOH	BF_3_•OEt_2_ (3)	RT, 12	69 (8.1:1)
16	AcOH	BF_3_•OEt_2_ (3)	RT, 6	71 (4.6:1)
17	AcOH	BF_3_•OEt_2_ (3)	RT, 2	68 (2.6:1)
18	AcOH	BF_3_•OEt_2_ (3)	RT, 48	68 (11.2:1)
19	AcOH	BF_3_•OEt_2_ (3)	50, 24	60 (10.9:1)
20	CH_2_Cl_2_	—	RT, 24	Trace
21	CH_2_Cl_2_	BF_3_•OEt_2_ (3)	RT, 24	23 (5.6:1)
22	CH_2_Cl_2_	BF_3_•OEt_2_ (3)/AcOH (10)	RT, 24	63 (9.3:1)
23	AcOEt	BF_3_•OEt_2_ (3)	RT, 24	48 (6.7:1)
24	AcOEt	BF_3_•OEt_2_ (3)/AcOH (10)	RT, 24	61 (8.2:1)
25	Et_2_O	BF_3_•OEt_2_ (3)	RT, 24	28 (3.5:1)
26	Et_2_O	BF_3_•OEt_2_ (3)/AcOH (10)	RT, 24	70 (4.7:1)
27	toluene	BF_3_•OEt_2_ (3)	RT, 24	18 (3.2:1)
28	toluene	BF_3_•OEt_2_ (3)/AcOH (10)	RT, 24	37 (8.2:1)
29	CH_3_CN	BF_3_•OEt_2_ (3)	RT, 24	Trace
30	CH_3_CN	BF_3_•OEt_2_ (3)/AcOH (10)	RT, 24	12 (>20:1)
31	CF_3_CH_2_OH	BF_3_•OEt_2_ (3)/AcOH (10)	RT, 24	37 (2.8:1)
32	(CF_3_)_2_CHOH	BF_3_•OEt_2_ (3)/AcOH (10)	RT, 24	44 (8.6:1)

a *Reaction conditions: reaction of 4-phenylcyclohexanone (1 equiv) with PhI(OAc)_2_ (1.5 equiv) and additives (1~3 equiv or without) in the acetic acid solvent or other organic solvents at room temperature for 2~48 h*.

b *The yield and the cis/trans ratio were determined by NMR analysis*.

Having established a practical method for the selective α-acetoxylation of 4-phenylcyclohexanone by hybrid catalysis, we became interested in the substrate generality for diastereoselective α-acetoxylation of cyclic ketones with the aforementioned hybrid system: hypervalent iodine (III) reagents and BF_3_•OEt_2_. The results are summarized (Table 2). Firstly, we tested several substituents at C4 position of cyclohexanones to probe the versatility of this hybrid system. Switching phenylcyclohexanone to 4-*tert*-butylcyclohexanone resulted in a slight decrease in the yield to 57% with similar *cis*-selectivity (Table 2, entry 2). Further replacing the substituent to the dimethylphenylsilyl group resulted in a further decrease in both yield and selectivity (Table 2, entry 3). Extending the reaction time to 24 h, better diastereoselectivity (>9: 1) was observed by lowing the yield to 21%, presumably due to a decomposition of the starting material under the reaction conditions. In the case of 3-substituted cyclohexanones, good to high selectivities were observed (Table 2, entries 4 and 5).

**Table 2:** Substrate scope for diastereoselective α-acetoxylation of cyclic ketones with hypervalent iodine(III) reagents and BF_3_•O${\text{Et}}_{2}^{\text{a}}$.

**Entry**	**Substrate**	**% yield (dr ratio)**
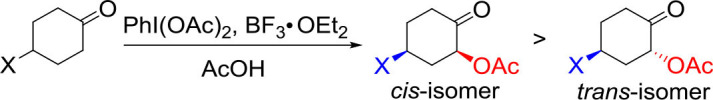		
1	X = Ph	*cis*-**1** 67 (11.8: 1)
2	X = *t*-Bu	*cis*-**2** 57 (11.1: 1)
3	X = –SiMe_2_Ph	*cis*-**3** 36^b^ (6.8: 1)
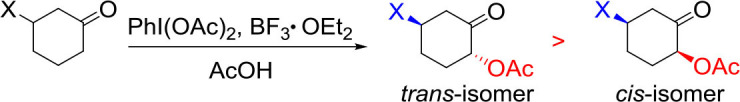		
4	X = Ph	*trans*-**4** 47 (6.1: 1)
5	X = *t*-Bu	*trans*-**5** 36 (12.3: 1)
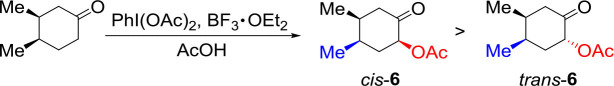		
6		*cis*-**6** 41^c^ (> 20: 1)
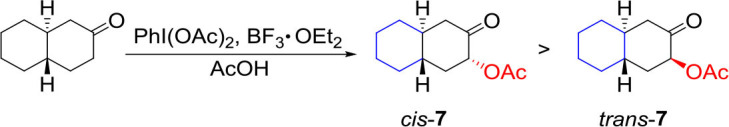		
7		*cis*-**7** 51 (9.9: 1)

a *Reaction conditions: reaction of cyclic ketone (1.0 equiv) with PhI(OAc)_2_ (1.5 equiv) and BF_3_•Et_2_O (3 equiv) in AcOH (1 mL) at room temperature for 24 h*.

b *The result was obtained when the reaction time was 0.5 h*.

c *The result was obtained when the reaction time was 3 h with 1.0 equiv BF_3_•Et_2_O. Standard reaction conditions provided 30% yield with dr > 20: 1*.

Thirdly, we studied the substrates with substituents at both C3 and C4 positions of cyclohexanones. *cis*-3,4-Dimethylcyclohexene as substrate gave 30% yield of the product *cis*-**6** with high diastereoselectivity (>20:1) under standard reaction conditions. Lowering the loading of BF_3_•OEt_2_ to 1.0 equiv resulted in a full consumption of starting material in 3 h with 41% yield and high diastereoselectivity (>20:1) (Table 2, entry 6). To our delight, *trans*-octahydro-naphthalen-2(1*H*)-one as substrate resulted in 51% yield of the product *cis*-**7** with 9.9: 1 diastereoselectivity under the reaction conditions (Table 2, entry 7). We tried the application of this hybrid system of PIDA and BF_3_•OEt_2_ to both cyclopentanone and cycloheptanone. Unfortunately, the yield of the target molecules were quiet low, indicating that this hybrid system was not effective for the α-acetoxylation of cyclopentanones or cycloheptanones.

**Scheme 2:**
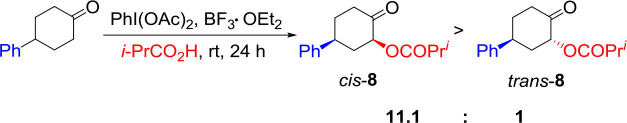
Synthesis of 2-acyloxy-4-phenylcyclohexanone **8** by using the hypervalent iodine (III) reagent–BF_3_•OEt_2_ hybrid system.

Having investigated the substrate scope for diastereoselective α-acetoxylation of cyclic ketones, we are interested in the application of the hypervalent iodine(III) reagent–BF_3_•OEt_2_ hybrid system. Changing AcOH solvent to *i*-PrCO_2_H, 2-acyloxy-4-phenylcyclohexanone **8** was obtained smoothly in 50% yield with high diastereoselectivity (11.1:1) (Scheme 2).

**Scheme 3:**
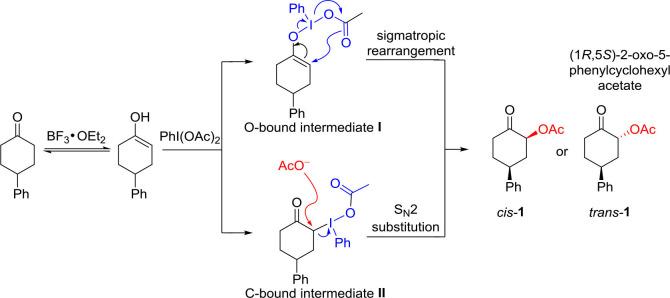
The possible mechanism for the hypervalent iodine-mediated α-acetoxylation of cyclic ketones.

It should be fundamentally interesting to understand the origin of the diastereoselectivity of the hypervalent iodine-mediated α-acetoxylation of cyclic ketones. With the assistance of Lewis acid BF_3_•OEt, the keto-enol equilibrium would lead to the activated enol to react with hypervalent iodine PhI(OAc)_2_. Generally, it is believed that an iodonium enolate (*O*-bound intermediate) or an α-iodanyl ketone (α-*C*-bound intermediate) could be formed for the subsequent α-acetoxylation. The *O*-bound hypervalent iodine intermediate can be traced back to Mizukami's proposal in their work in tosylation reactions in 1978. (Mizukami et al., 1978) The *C*-bound hypervalent iodine intermediate was first proposed by Moriarty et al. (1981). Accordingly, the possible mechanism for hypervalent iodine-mediated α-acetoxylation of cyclic ketones is proposed (Scheme 3). The *O*-bound intermediate would lead to the α-acetoxylation via the sigmatropic rearrangement pathway, while the *C*-bound intermediate will undergo an S_N_2 substitution by the acetate in the solution to form α-acetoxylation product.

To further understand the diastereoselectivity α-acetoxylation of cyclic ketones, density functional theory (DFT) calculations were carried out with Gaussian 16 program. Frisch et al. (2016) Geometry optimizations and frequency calculations were carried out using the M06-2X functional (Zhao and Truhlar, 2007; Walker et al., 2013) in solution by the SMD continuum solvent model (solvent = acetic acid) (Marenich et al., 2009), with the basis sets of SDD (Andrae et al., 1991) for iodine atom and 6–311++G^**^ for other atoms.

**Scheme 4:**
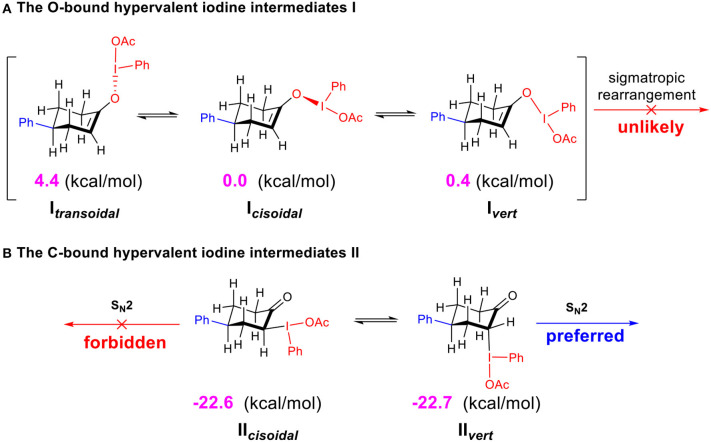
Relative free energies (in kcal/mol) of the *O*-bound intermediates I and *C*-bound intermediates II calculated at the M06-2X/6311++G^**^&SDD(I)/SMD(solvent = acetic acid) level of theory. **(A)** The *O*-bound hypervalent iodine intermediates I. **(B)** The *C*-bound hypervalent iodine intermediates II.

The calculated free energies of the hypervalent iodine intermediates **I** (*O*-bound) and **II** (*C*-bound) are depicted (Scheme 4). In the stable chair-conformer of 4-phenylcyclohexanone, different isomeric intermediates **I** (**I**_**transoidal**_, **I**_**cisoidal**_, and **I**_**vert**_) are calculated to be close in free energy, among which **I**_cisoidal_ is predicted to be the most stable one. In contrast, the *C*-bound intermediates **II** (**II**_**cisoidal**_ and **II**_**vert**_) are much more thermodynamically stable than the *O*-bound ones (by 22.6 (7) kcal/mol) in acetic acid. DFT results interestingly revealed a case of preferred *C*-bound species for the interaction between hypervalent iodine with cyclo-enol, which is complementary to previous suggestions that the enolate-like *O*-bound intermediates were more likely to be formed in the reaction of non-cyclic ketones. Norrby et al. (2010); Beaulieu and Legault (2015); Shneider et al. (2015); Arava et al. (2017) This predicted preference of α-*C*-bound hypervalent iodine species is also interestingly supported by the experimentally observed structures of the isolable iodonium ylides (Ivanov et al., 2014).

Since the *O*-bound intermediate **I** is more than 22 kcal/mol higher than the *C*-bound intermediate **II** in free energy, the sigmatropic rearrangement reaction pathway should be unlikely to operate in this system. The α-acetoxylation of cyclic ketones should prefer the S_N_2 substitution mechanism via the α-*C*-bound intermediate. This explains well the importance of acetic acid as solvent to facilitate the S_N_2 substitution in our studied system (Table 1, entries 20–27).

**Scheme 5:**
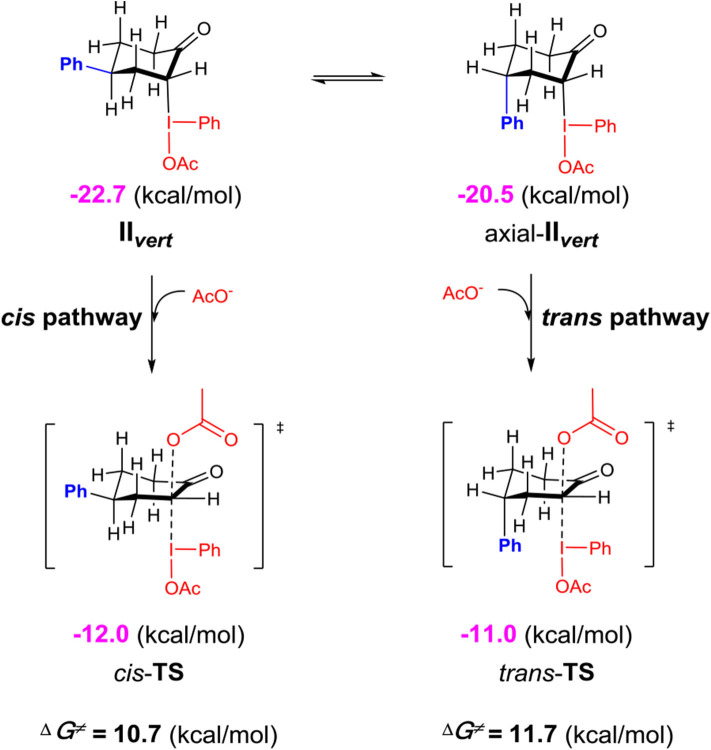
Relative free energies (in kcal/mol) of the isomers of α-*C*-bound intermediates II_vert_ calculated at the M06-2X/6311++G^**^&SDD(I)/SMD(solvent = acetic acid) level of theory.

**Scheme 6:**
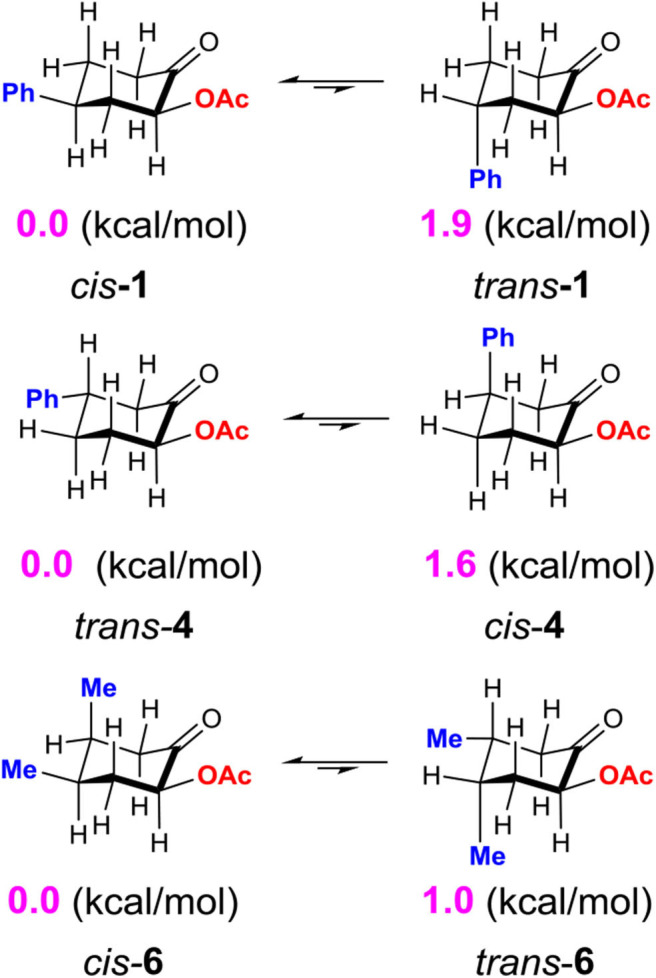
Relative free energy difference (in kcal/mol) between *cis* product and *trans* product for representative cyclic ketones, calculated at the M06-2X/6311++G^**^/SMD (solvent = acetic acid) level of theory.

Although the *C*-bound intermediates IIcisoidal and IIvert are close in free energy, the equatorial iodanyl group (IIcisoidal) is forbidden for S_N_2 substitution. The axial iodanyl intermediate IIvert should play a key role in the diastereoselective α-acetoxylation of cyclic ketones. Relative free energies (in kcal/mol) of the isomers of α-*C*-bound intermediates IIvert are depicted (Scheme 5). The equatorial phenyl intermediate IIvert corresponds to the *cis* product for the S_N_2 substitution, while the axial phenyl intermediate axial-IIvert leads to the *tran*s product. IIvert is thermodynamically more stable than axial-IIvert by 2.2 kcal/mol, due to the steric effect of the axial phenyl on the chair conformation of the cyclic ketone. According to the Hammond–Leffler postulate, this implies the kinetic preference of the formation of the *cis* product from intermediate IIvert. Indeed, the calculated free energy of the transition state (*cis*-TS) for the *cis* pathway is lower than that (*trans*-TS) for the *trans* Pathway by 1.0 kcal/mol. The free energy of *cis*-TS is −12.0 kcal/mol and the activation free energy of the S_N_2 step is only 10.7 kcal/mol, which further supports that the S_N_2 substitution mechanism should be more plausible than the sigmatropic rearrangement mechanism via the *O*-bound intermediate. More importantly, our experimentally observed diastereoselective results strongly supported the influence of the thermodynamic control in selectivity. Extending the reaction time from 2 to 48 h, the *cis/trans* ratio increased from 2.6:1 to 11.2:1, with unchanged yield (Table 1, entries 15–18), when we performed a control experiment by subjecting the product 1 with low cis/trans ratio of 3.1:1 to the standard reaction condition for 24 h, a cis/trans ratio of 11.8:1 was observed. The equilibrium between the *cis/trans* products in acetic acid was observed, leading to the most table isomer as the major product. To further verify the thermodynamic control selectivity, we compared the relative free energy difference between *cis* product and *trans* product for representative substrates, i.e., 4-phenylcyclohexanone, 3-phenylcyclohexanone, and *cis*-3,4-dimethylcyclohexene, as shown in Scheme 6. The DFT-predicted free energy difference clearly demonstrated the thermodynamic control in selectivity, which is in good agreement with the experimentally observed diastereoselective ratio. The product *cis*-**1** is lower in free energy than *trans*-**1** by 1.9 kcal/mol, consistent with the experimentally observed *cis/trans* ratio of 11.2:1. When the phenyl substituent moves from the β position of the acetoxyl to the further γ position, the free energy difference decreases to 1.6 kcal/mol, in accordance with a slightly lower diastereoselective ratio of 6.1:1, and the inverse ratio of *trans/cis* is well-reproduced. With respect to *cis*-3,4-dimethylcyclohexene, DFT results suggest a free energy difference of 1.0 kcal/mol for the *cis/trans* products, in good agreement with the experimentally observed *cis/trans* ratio of 6.8:1 as well-indicating the β-substituent is predominant compared to the γ-position, probably due to the distance between the substituent and the acetoxyl group.

## Conclusion

In conclusion, we have developed a practical approach for diastereoselective α-acetoxylation of cyclic ketones by a binary hybrid system comprising a hypervalent iodine(III) reagent and BF_3_•OEt_2_ Lewis acid. In this hybrid system, BF_3_•OEt_2_ Lewis acid allowed the activation of the hypervalent iodine(III) reagent for the smooth α-acetoxylation reaction of cyclic ketones and also for achieving high diastereoselectivity. The substrate scoping investigation showed that sterically hindered substituent groups on cyclic ketones are favorable for the diastereoselectivity. Cyclic ketone substrates bearing the mono-substituent group at C3 or C4 position, and substrates bearing *di*-substituent groups at C3 and C4 positions demonstrated good to high diastereoselectivity. This approach was successfully applied to the synthesis of other *cis*-substituted 2-acyloxycyclohexanones in moderate yield with high diastereoselectivity. Computational studies showed that this α-acetoxylation of cyclic ketone reaction plausibly undergoes an S_N_2 substitution mechanism, in which the α-*C*-bound hypervalent iodine species is the important intermediate. The diastereoselectivity of the reaction mainly originated from the thermodynamic control. This hypervalent iodine(III) reagent–BF_3_•OEt_2_ hybrid system can thus be applied to certain selective organic reactions and the efficient diastereoselective synthesis of cyclic ketone derivatives or other related biologically active compounds.

## Materials and Methods

### General

^1^H NMR and ^13^C NMR spectra were recorded on a Bruker AVANCE III 400 MHz spectrometer [400 MHz for ^1^H NMR, 100 MHz for ^13^C NMR]. Tetramethylsilane (TMS) was used as an internal standard (0 ppm) for the ^1^H NMR spectra, and CDCl_3_ was used as the internal standard (77.0 ppm) for the ^13^C NMR spectra. High-resolution mass spectra (HRMS) were recorded on a Thermo MAT95XP or on an Agilent 6540 UHD Accurate-Mass Q-TOF LC-MS spectrometer. Infrared (IR) spectra were obtained on a Thermo Scientific Nicolet FT/IR-6700 spectrometer. Reactions were monitored by thin-layer chromatography (TLC). Reaction products were purified by column chromatography on silica gel. AcOH was dried before use. Other chemical reagents were purchased from common commercial suppliers and used as received.

General procedures for the α-acetoxylation of cyclic ketones: To a solution of cyclic ketones (0.5 mmol) and PhI(OAc)_2_ (241.5 mg, 0.75 mmol) in acetic acid (1 mL) was added BF_3_•OEt_2_ (212.9 mg, 1.5 mmol) dropwise, and the reaction mixture was stirred at room temperature for 24 h. The reaction progress was monitored by TLC. Upon completion, the reaction mixture was quenched with saturated aqueous Na_2_S_2_O_3_ and then saturated aqueous NaHCO_3_, washed with brine, extracted with dichloromethane, and dried over anhydrous Na_2_SO_4_. After filtration, the solvent was removed under reduced pressure to afford the crude product, which was purified by silica gel column chromatography using hexane/acetone and analyzed by ^1^H and ^13^C NMR spectroscopy.

#### 2-Oxo-5-Phenylcyclohexyl Acetate (1)

The crude product was purified via flash chromatography, eluting with hexane/acetone = 30/1 to give a white solid (77.8 mg, 67%). Characterization of the major product (*cis*-isomer): Mp: 90–92°C; ^1^H NMR (400 MHz, CDCl_3_): δ = 7.35–7.23 (m, 5H), 5.38 (dd, *J* = 12.8, 6.4 Hz, 1H), 3.23 (t, *J* = 12.4 Hz, 1H), 2.62–2.59 (m, 2H), 2.49–2.44 (m, 1H), 2.27–2.24 (m, 1H), 2.17 (s, 3H), 2.08 (q, *J* = 12.4 Hz, 1H), 1.96–1.85 (m, 1H); ^13^C NMR (100 MHz, CDCl_3_): δ = 204.0, 170.1, 143.2, 128.9, 127.1, 126.8, 75.8, 42.1, 40.0, 39. 9, 34.5, 20.8; IR (KBr): 3,081, 3,021, 2,938, 2,875, 2,854, 1,757, 1,718, 1,641, 1,492, 1,445, 1,423, 1,385, 1,370, 1,324, 1,287, 1,263, 1,245, 1,150, 1,120, 1,072, 1,061, 982, 935, 911, 852, 756, 743, 695, 610, 517, 466 cm^−1^; HRMS (ESI) calcd for C_14_H_17_O_3_ [M+H]^+^: 233.1178; found: 233.1168; calcd for C_14_H_16_O_3_Na [M+Na]^+^: 255.0997; found: 255.0987.

#### 5-(tert-Butyl)-2-Oxocyclohexyl Acetate (2)

The crude product was purified via flash chromatography, eluting with hexane/acetone = 30/1 to give a colorless oil (60.5 mg, 57%). Characterization of the major product (*cis*-isomer): ^1^H NMR (400 MHz, CDCl_3_): δ = 5.23–5.18 (m, 1H), 2.52–2.47 (m, 1H), 2.43–2.34 (m, 1H), 2.33–2.27 (m, 1H), 2.15 (s, 3H), 2.13–2.07 (m, 1H), 1.74–1.66 (m, 1H), 1.57 (q, *J* = 12.4 Hz, 1H), 1.48–1.37 (m, 1H), 0.93 (s, 9H); ^13^C NMR (100 MHz, CDCl_3_): δ = 205.0, 170.2, 76.3, 46.0, 39.7, 34.4, 32.6, 28.2, 27.7, 20.9.

#### 5-(Dimethyl(Phenyl)Silyl)-2-Oxocyclohexyl Acetate (3)

The crude product was purified via flash chromatography, eluting with hexane/acetone = 30/1 to give a light yellow solid (52.3 mg, 36%). Characterization of the major product (*cis*-isomer): Mp: 121–123°C; ^1^H NMR (400 MHz, CDCl_3_): δ = 7.49–7.37 (m, 5H), 5.15 (dd, *J* = 12.4, 6.4 Hz, 1 H), 2.53–2.50 (m, 1H), 2.43–2.35 (m, 1H), 2.27–2.23 (m, 1H), 2.13 (s, 3H), 2.09–2.04 (m, 1H), 1.61 (q, *J* = 12.8 Hz, 1H), 1.53–1.42 (m, 1H), 1.38–1.32 (m, 1H), 0.33 (s, 6H); ^13^C NMR (100 MHz, CDCl_3_): δ = 204.9, 170.2, 136.5, 133.9, 129.9, 129.6, 128.1, 77.7, 42.5, 34.7, 28.9, 24.1, 20.9, −4.9, −5.0; IR (KBr): 3,071, 3,012, 2,949, 2,935, 2,865, 2,841, 1,748, 1,721, 1,427, 1,407, 1,376, 1,342, 1,321, 1,257, 1,233, 1,173, 1,143, 1,112, 1,102, 1,082, 1,050, 968, 912, 885, 850, 834, 821, 776, 763, 742, 728, 704, 661, 643, 605, 570, 482, 451, 436 cm^−1^; HRMS (ESI) calcd for C_16_H_23_O_3_Si [M+H]^+^: 291.1416; found: 291.1420; calcd for C_16_H_22_O_3_Na [M+Na]^+^: 313.1236; found: 313.1227.

#### 2-Oxo-4-Phenylcyclohexyl Acetate (4)

The crude product was purified via flash chromatography, eluting with hexane/acetone = 30/1 to give a white solid (54.6 mg, 47%). Characterization of the major product (*trans*-isomer): Mp: 72–74°C; ^1^H NMR (400 MHz, CDCl_3_): δ = 7.35–7.31 (m, 2H), 7.25–7.20 (m, 3H), 5.29 (dd, *J* = 12.8, 6.4 Hz, 1H), 3.02–2.94 (m, 1H), 2.70–2.62 (m, 2H), 2.40–2.35 (m, 1H), 2.18 (s, 3H), 2.14 (m, 1H), 2.09–1.98 (m, 1H), 1.95–1.84 (m, 1H); ^13^C NMR (100 MHz, CDCl_3_): δ = 203.4, 170.3, 143.2, 129.0, 127.2, 126.6, 76.3, 47.9, 45.4, 31.8, 31.7, 20.9; IR (KBr): 3,033, 2,959, 2,941, 2,908, 1,745, 1,721, 1,602, 1,501, 1,458, 1,432, 1,376, 1,319, 1,281, 1,233, 1,174, 1,081, 1,046, 897, 763, 703, 665, 599, 531, 501 cm^−1^; HRMS (ESI) calcd for C_14_H_17_O_3_ [M+H]^+^: 233.1178; found: 233.1145; calcd for C_14_H_16_O_3_Na [M+Na]^+^: 255.0997; found: 255.0986.

#### 4-(Tert-Butyl)-2-Oxocyclohexyl Acetate (5)

The crude product was purified via flash chromatography, eluting with hexane/acetone = 30/1 to give a light yellow oil (38.2 mg, 36%). Characterization of the major product (*trans*-isomer): ^1^H NMR (400 MHz, CDCl_3_): δ = 5.15 (dd, *J* = 12.8, 6.8 Hz, 1H), 2.56–2.53 (m, 1H), 2.33–2.27 (m, 1H), 2.22–2.13 (m, 1H), 2.16 (s, 3H), 2.03–2.00 (m, 1H), 1.73–1.63 (m, 1H), 1.56–1.51 (m, 2H), 0.91 (s, 9H); ^13^C NMR (100 MHz, CDCl_3_): δ = 205.3, 170.3, 76.6, 50.0, 42.4, 32.9, 31.7, 27.4, 25.0, 20.9.

#### 4,5-Dimethyl-2-Oxocyclohexyl Acetate (6)

The crude product was purified via flash chromatography, eluting with hexane/acetone = 30/1 to give a light yellow oil (37.8 mg, 41%). Characterization of the major isomer: ^1^H NMR (400 MHz, CDCl_3_): δ = 5.19 (dd, *J* = 12.4, 6.8 Hz, 1H), 2.67–2.62 (m, 1H), 2.35–2.21 (m, 3H), 2.14 (s, 3H), 2.07–2.03 (m, 1H), 1.74 (q, *J* = 12.8 Hz, 1H), 1.01 (d, *J* = 6.8 Hz, 3H), 0.83 (d, *J* = 6.8 Hz, 3H); ^13^C NMR (100 MHz, CDCl_3_): δ = 204.6, 170.1, 75.6, 47.3, 36.5, 34.9, 33.2, 20.8, 18.5, 12.1; IR (KBr): 2,959, 2,928, 2,891, 2,871, 1,749, 1,721, 1,470, 1,455, 1,431, 1,380, 1,370, 1,243, 1,175, 1,102, 1,087, 1,075, 1,036, 975, 941, 885, 790, 715, 651, 609, 549, 510, 482, 436 cm^−1^; HRMS (ESI) calcd for C_10_H_16_O_3_Na [M+Na]^+^: 207.0997; found: 207.0988.

#### 3-Oxodecahydronaphthalen-2-yl Acetate (7)

The crude product was purified via flash chromatography, eluting with hexane/acetone = 30/1 to give a light yellow solid (53.6 mg, 51%). Characterization of the major isomer: ^1^H NMR (400 MHz, CDCl_3_): δ = 5.19 (dd, *J* = 12.0, 6.8 Hz, 1H), 2.41–2.37 (m, 1H), 2.20–2.10 (m, 2H), 2.13 (s, 3H), 1.78–1.68 (m, 4H), 1.56–1.46 (m, 2H), 1.36–0.99 (m, 5H); ^13^C NMR (100 MHz, CDCl_3_): δ = 204.0, 170.2, 76.1, 47.2, 43.8, 40.5, 39.4, 33.7, 32.5, 25.8, 25.5, 20.8.

#### 2-Oxo-5-Phenylcyclohexyl Isobutyrate (8)

The crude product was purified via flash chromatography, eluting with hexane/acetone = 30/1 to give a white solid (65.1 mg, 50%). Characterization of the major isomer: Mp: 63–65°C; ^1^H NMR (400 MHz, CDCl_3_): δ = 7.38–7.34 (m, 2H), 7.29–7.26 (m, 3H), 5.41(dd, *J* = 12.8, 6.0 Hz, 1H), 3.26 (t, *J* = 12.8 Hz, 1H), 2.71–2.62 (m, 3H), 2.51–2.46 (m, 1H), 2.30–2.26 (m, 1H), 2.11 (q, *J* = 12.8 Hz, 1H), 2.00–1.89 (m, 1H); 1.28 (d, *J* = 6.8 Hz, 3H), 1.23 (d, *J* = 6.8 Hz, 3H); ^13^C NMR (100 MHz, CDCl_3_): δ = 204.1, 176.4, 143.3, 128.9, 127.1, 126.8, 75.4, 42.1, 40.0, 39.9, 34.5, 34.0, 19.2, 19.1; IR (KBr): 3,030, 2,977, 2,929, 2,866, 1,751, 1,727, 1,632, 1,605, 1,498, 1,462, 1,429, 1,385, 1,349, 1,293, 1,260, 1,200, 1,165, 1,147, 1,117, 1,069, 977, 918, 843, 763, 739, 701, 596, 540, 507 cm^−1^; HRMS (ESI) calcd for C_16_H_20_O_3_Na [M+Na]^+^: 283.1310; found: 283.1299.

## Data Availability Statement

The datasets presented in this study can be found in online repositories. The names of the repository/repositories and accession number(s) can be found below: Cambridge Crystallographic Data Centre (CCDC-1997827).

## Author Contributions

JT, WZ, and WX were responsible for designing and performing the experiments. YJ and ZK were responsible for DPT calculation. YL and KM directed the project and wrote the manuscript.

## Conflict of Interest

The authors declare that the research was conducted in the absence of any commercial or financial relationships that could be construed as a potential conflict of interest.
