# Supplementary material for: Hypervalent Iodine-Mediated Diastereoselective α-Acetoxylation of Cyclic Ketones
Source: Front Chem. 2020 Jul 10;8:467. doi: 10.3389/fchem.2020.00467 (PMC7365914; doi:10.3389/fchem.2020.00467)
Supplement: Supplementary file 1 [file Data_Sheet_1.pdf]

Supplementary Material

Hypervalent Iodine Mediated Diastereoselective α-Acetoxylation of Cyclic Ketones

Jiashen Tan[#1], Weiqin Zhu[#1], Weiping Xu[1], Yaru Jing[1,2], Zhuofeng Ke[2], Yan Liu*[1], Keiji Maruoka*[1,3]

Table of contents

S. No.	Section	Page No.
1.	General	S2
2.	General experimental procedure for the reaction	S2
3.	Optimization of reaction conditions	S3-S4
4.	Spectral data for products (**1-8**)	S5-S7
5.	X-Ray Diffraction Analysis of compound *cis*-**1**	S7
6.	Copies of ^{1}H and ^{13}C NMR spectra of products	S8-S15
7	Computational details and references	S16
8	Cartesian coordinates the optimized structures	S17-S27

General

^{1}H NMR and ^{13}C NMR spectra were recorded on a Bruker AVANCE III 400 MHz spectrometer (400 MHz for ^{1}H NMR, 100 MHz for ^{13}C NMR). Tetramethylsilane (TMS) was used as an internal standard (0 ppm) for the ^{1}H NMR spectra, and $CDCl_3$ was used as the internal standard (77.16 ppm) for the ^{13}C NMR spectra. Multiplicity is indicated as follows: s (singlet); bs (broad singlet); d (doublet); t (triplet); q (quartet); m (multiplet); dd (doublet of doublets), etc. High-resolution mass spectra (HRMS) were recorded on a Thermo MAT95XP, or on an Agilent 6540 UHD Accurate-Mass Q-TOF LC-MS spectrometer. Infrared (IR) spectra were obtained on a Thermo scientific Nicolet FT/IR-6700 spectrometer. Reactions were monitored by thin-layer chromatography (TLC). Reaction products were purified by column chromatography on silica gel. AcOH was dried before use. Other chemical reagents were purchased from common commercial suppliers and used as received.

General procedures for the α-acetoxylation of cyclic ketones

To a solution of cyclic ketones (0.5 mmol) and $PhI(OAc)_2$ (241.5 mg, 0.75 mmol) in acetic acid (1 mL) was added $BF_3{\bullet}OEt_2$ (212.9 mg ,1.5 mmol) dropwise, and the reaction mixture was stirred at room temperature for 24 h. The reaction progress was monitored by TLC. Upon completion, the reaction mixture was quenched with saturated aqueous $Na_2S_2O_3$ and then saturated aqueous $NaHCO_3$, washed with brine, extracted with dichloromethane, and dried over anhydrous Na_2SO_4. After filtration, the solvent was removed under reduced pressure to afford the crude product, which was purified by silica gel column chromatography using hexane/acetone and analyzed by ^{1}H and ^{13}C NMR spectroscopy.

TABLE 1 Diastereoselective α-acetoxylation of ketone with hypervalent iodine (III) reagents in the presence of additives.[a]

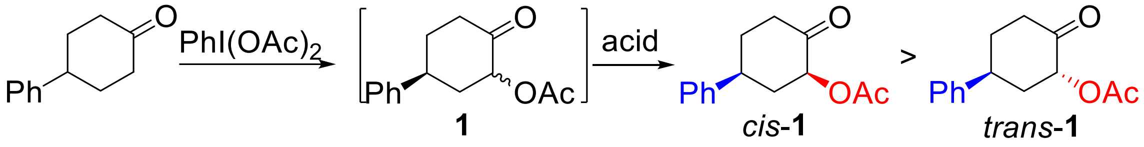

entry	solvent	additive (equiv)	condition (°C, h)	% yield of **1**[b] (*cis/trans* ratio)
1	AcOH	—	RT, 24	trace
2	AcOH	CF_3CO_2H (1)	RT, 24	17 (1.4 : 1)
3	AcOH	TfOH (1)	RT, 24	54 (12.2 : 1)
4	AcOH	AgOTf (1)	RT, 24	37 (2.1 : 1)
5	AcOH	$ZnCl_2$ (1)	RT, 24	0
6	AcOH	$AlCl_3$ (1)	RT, 24	0
7	AcOH	$Sc(OTf)_3$ (1)	RT, 24	42 (7.0 : 1)
8	AcOH	$Mg(OTf)_2$ (1)	RT, 24	52 (2.1 : 1)
9	AcOH	$Cu(OTf)_2$ (1)	RT, 24	60 (3.7 : 1)
10	AcOH	$Zn(OTf)_2$ (1)	RT, 24	68 (3.0 : 1)
11	AcOH	BF_3•OEt_2 (1)	RT, 24	70 (6.8 : 1)
12	AcOH	BF_3•OEt_2 (2)	RT, 24	73 (10.5 : 1)
13	AcOH	BF_3•OEt_2 (3)	RT, 24	67 (11.8 : 1)
14	AcOH	BF_3•OEt_2 (5)	RT, 24	71 (12.5 : 1)
15	AcOH	BF_3•OEt_2 (3)	RT, 12	69 (8.1 : 1)
16	AcOH	BF_3•OEt_2 (3)	RT, 6	71 (4.6 : 1)
17	AcOH	BF_3•OEt_2 (3)	RT, 2	68 (2.6 : 1)
18	AcOH	BF_3•OEt_2 (3)	RT, 48	68 (11.2 : 1)

19	AcOH	BF_3•OEt_2 (3)	50, 24	60 (10.9 : 1)
20	CH_2Cl_2	——	RT, 24	trace
21	CH_2Cl_2	BF_3•OEt_2 (3)	RT, 24	23 (5.6 : 1)
22	CH_2Cl_2	BF_3•OEt_2 (3)/AcOH (10)	RT, 24	63 (9.3 : 1)
23	AcOEt	BF_3•OEt_2 (3)	RT, 24	48 (6.7 : 1)
24	AcOEt	BF_3•OEt_2 (3)/AcOH (10)	RT, 24	61 (8.2 : 1)
25	Et_2O	BF_3•OEt_2 (3)	RT, 24	28 (3.5 : 1)
26	Et_2O	BF_3•OEt_2 (3)/AcOH (10)	RT, 24	70 (4.7 : 1)
27	toluene	BF_3•OEt_2 (3)	RT, 24	18 (3.2 : 1)
28	toluene	BF_3•OEt_2 (3)/AcOH (10)	RT, 24	37 (8.2 : 1)
29	CH_3CN	BF_3•OEt_2 (3)	RT, 24	trace
30	CH_3CN	BF_3•OEt_2 (3)/AcOH (10)	RT, 24	12 (>20 : 1)
31	CF_3CH_2OH	BF_3•OEt_2 (3)/AcOH (10)	RT, 24	37 (2.8 : 1)
32	$(CF_3)_2CHOH$	BF_3•OEt_2 (3)/AcOH (10)	RT, 24	44 (8.6 : 1)

[a]Reaction conditions: reaction of 4-phenylcyclohexanone (1 equiv) with $PhI(OAc)_2$ (1.5 equiv) and additives (1~3 equiv or without) in acetic acid solvent or other organic solvents at room temperature for 2~48 h. [b]The yield and the *cis/trans* ratio were determined by NMR analysis.

2-Oxo-5-phenylcyclohexyl acetate (1): The crude product was purified via flash chromatography, eluting with hexane/acetone = 30/1 to give a white solid (77.8 mg, 67%). Characterization of the major product (*cis*-isomer): Mp: 90–92 ºC; ^{1}H NMR (400 MHz, $CDCl_3$): δ = 7.35-7.23 (m, 5H), 5.38 (dd, *J* = 12.8, 6.4 Hz, 1H), 3.23 (t, *J* = 12.4 Hz, 1H), 2.62-2.59 (m, 2H), 2.49-2.44 (m, 1H), 2.27-2.24 (m, 1H), 2.17 (s, 3H), 2.08 (q, *J* = 12.4 Hz, 1H), 1.96-1.85 (m, 1H); ^{13}C NMR (100 MHz, $CDCl_3$): δ = 204.0, 170.1, 143.2, 128.9, 127.1, 126.8, 75.8, 42.1, 40.0, 39. 9, 34.5, 20.8; IR (KBr): 3081, 3021, 2938, 2875, 2854, 1757, 1718, 1641, 1492, 1445, 1423, 1385, 1370, 1324, 1287, 1263, 1245, 1150, 1120, 1072, 1061, 982, 935, 911, 852, 756, 743, 695, 610, 517,466 cm^{-1}; HRMS (ESI) calcd for $C_{14}H_{17}O_3$ $[M+H]^+$: 233.1178; found: 233.1168; calcd for $C_{14}H_{16}O_3Na$ $[M+Na]^+$: 255.0997; found: 255.0987.

5-(tert-Butyl)-2-oxocyclohexyl acetate (2): The crude product was purified via flash chromatography, eluting with hexane/acetone = 30/1 to give a colorless oil (60.5 mg, 57%). Characterization of the major product (*cis*-isomer): ^{1}H NMR (400 MHz, $CDCl_3$): δ = 5.23-5.18 (m, 1H), 2.52-2.47 (m, 1H), 2.43-2.34 (m, 1H), 2.33-2.27 (m, 1H), 2.15 (s, 3H), 2.13-2.07 (m, 1H), 1.74-1.66 (m, 1H), 1.57 (q, *J* = 12.4 Hz, 1H), 1.48-1.37(m, 1H), 0.93 (s, 9H); ^{13}C NMR (100 MHz, $CDCl_3$): δ□= 205.0, 170.2 , 76.3, 46.0, 39.7, 34.4, 32.6, 28.2, 27.7, 20.9.

5-(Dimethyl(phenyl)silyl)-2-oxocyclohexyl acetate (3): The crude product was purified via flash chromatography, eluting with hexane/acetone = 30/1 to give a light yellow solid (52.3 mg, 36%). Characterization of the major product (*cis*-isomer): Mp: 121–123 ºC; ^{1}H NMR (400 MHz, $CDCl_3$): δ□= 7.49-7.37 (m, 5H), 5.15 (dd, *J* = 12.4, 6.4 Hz, 1 H), 2.53-2.50 (m, 1H), 2.43-2.35 (m, 1H), 2.27-2.23 (m, 1H), 2.13 (s, 3H), 2.09-2.04 (m, 1H), 1.61 (q, *J* = 12.8 Hz, 1H), 1.53-1.42 (m, 1H), 1.38-1.32 (m, 1H), 0.33 (s, 6H); ^{13}C NMR (100 MHz, $CDCl_3$): δ = 204.9, 170.2, 136.5, 133.9, 129.9, 129.6, 128.1, 77.7, 42.5, 34.7, 28.9, 24.1, 20.9, -4.9, -5.0; IR (KBr): 3071, 3012, 2949, 2935, 2865, 2841, 1748, 1721, 1427, 1407, 1376, 1342, 1321, 1257, 1233, 1173, 1143, 1112, 1102, 1082, 1050, 968, 912, 885, 850, 834, 821, 776, 763, 742, 728, 704, 661, 643, 605, 570, 482, 451, 436 cm^{-1}; HRMS (ESI) calcd for $C_{16}H_{23}O_3Si$ $[M+H]^+$: 291.1416; found: 291.1420; calcd for $C_{16}H_{22}O_3Na$ $[M+Na]^+$: 313.1236; found: 313.1227.

2-Oxo-4-phenylcyclohexyl acetate (4): The crude product was purified via flash chromatography, eluting with hexane/acetone = 30/1 to give a white solid (54.6 mg, 47%). Characterization of the major product (*trans*-isomer): Mp: 72–74 ºC; ^{1}H NMR (400 MHz, $CDCl_3$): δ□= 7.35-7.31 (m, 2H), 7.25-7.20 (m, 3H), 5.29 (dd, *J* = 12.8, 6.4 Hz, 1H), 3.02-2.94 (m, 1H), 2.70-2.62 (m, 2H), 2.40-2.35 (m, 1H), 2.18 (s, 3H), 2.14 (m, 1H), 2.09-1.98 (m, 1H), 1.95-1.84 (m, 1H); ^{13}C NMR (100 MHz, $CDCl_3$): δ = 203.4, 170.3, 143.2,

129.0, 127.2, 126.6, 76.3, 47.9, 45.4, 31.8, 31.7, 20.9; IR (KBr): 3033, 2959, 2941, 2908, 1745, 1721, 1602, 1501, 1458, 1432, 1376, 1319, 1281, 1233, 1174, 1081, 1046, 897, 763, 703, 665, 599, 531, 501 cm^{-1}; HRMS (ESI) calcd for $C_{14}H_{17}O_3$ $[M+H]^+$: 233.1178; found: 233.1145; calcd for $C_{14}H_{16}O_3Na$ $[M+Na]^+$: 255.0997; found: 255.0986.

4-(tert-Butyl)-2-oxocyclohexyl acetate (5): The crude product was purified via flash chromatography, eluting with hexane/acetone = 30/1 to give a light yellow oil (38.2 mg, 36%). Characterization of the major product (*trans*-isomer): ^1H NMR (400 MHz, $CDCl_3$): δ = 5.15 (dd, *J* = 12.8, 6.8 Hz, 1H), 2.56-2.53 (m, 1H), 2.33-2.27 (m, 1H), 2.22-2.13 (m, 1H), 2.16 (s, 3H), 2.03-2.00 (m, 1H), 1.73-1.63 (m, 1H), 1.56-1.51 (m, 2H), 0.91 (s, 9H); ^{13}C NMR (100 MHz, $CDCl_3$): δ = 205.3, 170.3, 76.6, 50.0, 42.4, 32.9, 31.7, 27.4, 25.0, 20.9.

4,5-Dimethyl-2-oxocyclohexyl acetate (6): The crude product was purified via flash chromatography, eluting with hexane/acetone = 30/1 to give a light yellow oil (37.8 mg, 41%). Characterization of the major isomer: ^1H NMR (400 MHz, $CDCl_3$): δ = 5.19 (dd, *J* = 12.4, 6.8Hz, 1H), 2.67-2.62 (m, 1H), 2.35-2.21 (m, 3H), 2.14 (s, 3H), 2.07-2.03 (m, 1H), 1.74 (q, *J* = 12.8 Hz, 1H), 1.01 (d, *J* = 6.8 Hz, 3H), 0.83 (d, *J* = 6.8 Hz, 3H); ^{13}C NMR (100 MHz, $CDCl_3$): δ = 204.6, 170.1, 75.6, 47.3, 36.5, 34.9, 33.2, 20.8, 18.5, 12.1; IR (KBr): 2959, 2928, 2891, 2871, 1749, 1721, 1470, 1455, 1431, 1380, 1370, 1243, 1175, 1102, 1087, 1075, 1036, 975, 941, 885, 790, 715, 651, 609, 549, 510, 482, 436 cm^{-1}; HRMS (ESI) calcd for $C_{10}H_{16}O_3Na$ $[M+Na]^+$: 207.0997; found: 207.0988.

3-Oxodecahydronaphthalen-2-yl acetate (7): The crude product was purified via flash chromatography, eluting with hexane/acetone = 30/1 to give a light yellow solid (53.6 mg, 51%). Characterization of the major isomer:^1H NMR (400 MHz, $CDCl_3$): δ = 5.19 (dd, *J* = 12.0, 6.8 Hz, 1H), 2.41-2.37 (m, 1H), 2.20-2.10 (m, 2H), 2.13 (s, 3H), 1.78-1.68 (m, 4H), 1.56-1.46 (m, 2H), 1.36-0.99 (m, 5H); ^{13}C NMR (100 MHz, $CDCl_3$): δ = 204.0, 170.2, 76.1, 47.2, 43.8, 40.5, 39.4, 33.7, 32.5, 25.8, 25.5, 20.8.

2-Oxo-5-phenylcyclohexyl isobutyrate (8): The crude product was purified via flash chromatography, eluting with hexane/acetone = 30/1 to give a white solid (65.1 mg, 50%). Characterization of the major isomer: Mp: 63–65 ºC; ^1H NMR (400 MHz, $CDCl_3$): δ = 7.38-7.34 (m, 2H), 7.29-7.26 (m, 3H), 5.41(dd, *J* = 12.8, 6.0 Hz, 1H), 3.26 (t, *J* = 12.8 Hz, 1H), 2.71-2.62 (m, 3H), 2.51-2.46 (m, 1H), 2.30-2.26 (m, 1H), 2.11 (q, *J* = 12.8 Hz, 1H), 2.00-1.89 (m, 1H); 1.28 (d, *J* = 6.8 Hz, 3H), 1.23 (d, *J* = 6.8 Hz, 3H); ^{13}C NMR (100 MHz, $CDCl_3$): δ = 204.1, 176.4, 143.3, 128.9, 127.1, 126.8, 75.4, 42.1, 40.0, 39.9, 34.5, 34.0, 19.2, 19.1; IR

(KBr): 3030, 2977, 2929, 2866, 1751, 1727, 1632, 1605, 1498, 1462, 1429, 1385, 1349, 1293, 1260, 1200, 1165, 1147, 1117, 1069, 977, 918, 843, 763, 739, 701, 596, 540, 507 cm^{-1}; HRMS (ESI) calcd for $C_{16}H_{20}O_3Na$ $[M+Na]^+$: 283.1310; found: 283.1299.

X-Ray Diffraction Analysis of compound *cis*-1

Compound *cis*-**1** was recrystalized from ethyl acetate/hexane. Single crystal X-ray diffraction data were collected on an XtaLAB Synergy R, DW system, HyPix diffractometer with Cu Kα (λ = 1.54184 Å) radiation. The crystal was kept at 100.01(10) K during data collection. The crystal structure was solved with the ShelXD structure solution program (Sheldrick, 2008), and refined by full-matrix least-squares with the ShelXL (Sheldrick, 2015) in Olex 2-1.3 (Dolomanov et al., 2009).

The crystallographic data of compound *cis*-**1** were summarized in the following table

empirical formula	$C_{14}H_{16}O_3$
formula weight	232.27
cystal system	monoclinic
space group	$P2_1/n$
a, Å	6.3213 (2)
b, Å	30.3013 (7)
c, Å	6.9028 (3)
V, $Å^3$	1191.22 (7)
Z	4
dcalc, g/cm^3	1.295
T, K	100.01 (10)
R_1 ($I > 2\sigma(I)$)	0.0517
wR_2 (all data)	0.1400
Goodness of Fit	1.043

The crystal structure has been deposited at the Cambrige Crystallographic Data Centre (CCDC-1997827). The data can be obtained free of charge via the Internet at www.ccdc.cam.ac.uk/data_request/cif.

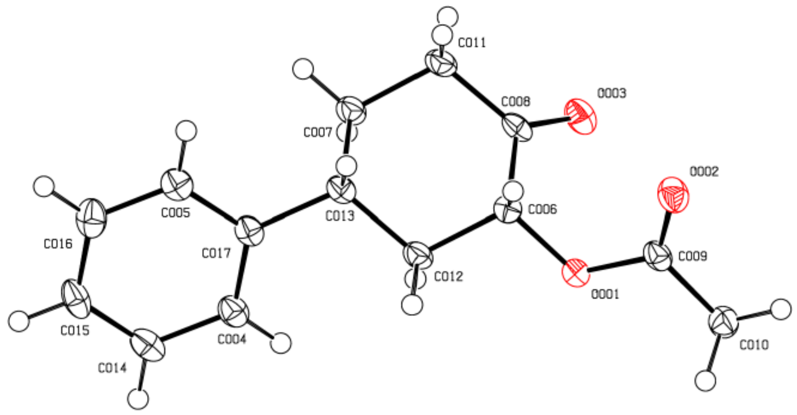

Copies of ^{1}H and ^{13}C NMR spectra of products:

^{1}H and ^{13}C NMR spectra of product *cis*-1

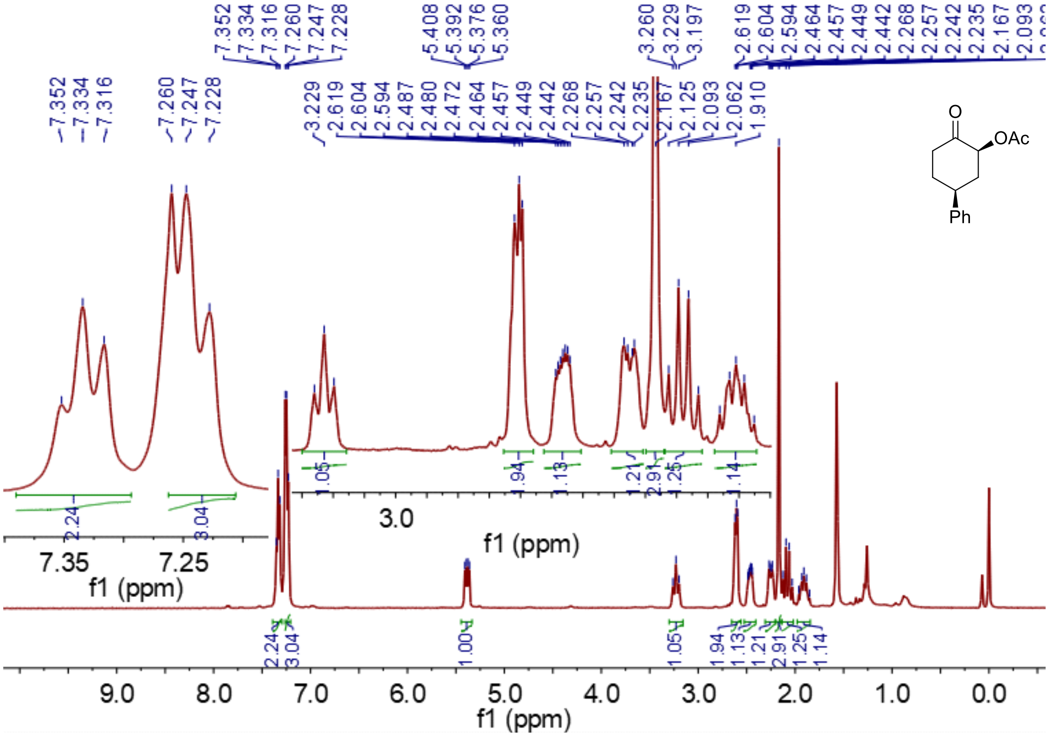

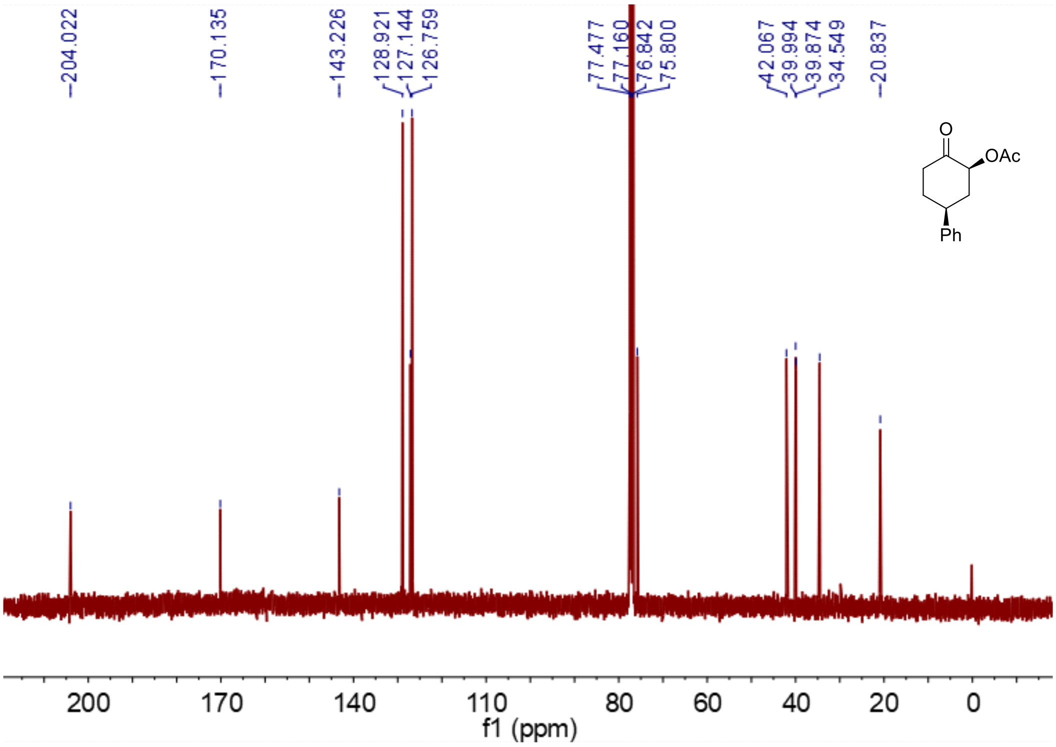

^{1}H and ^{13}C NMR spectra of product *cis*-2

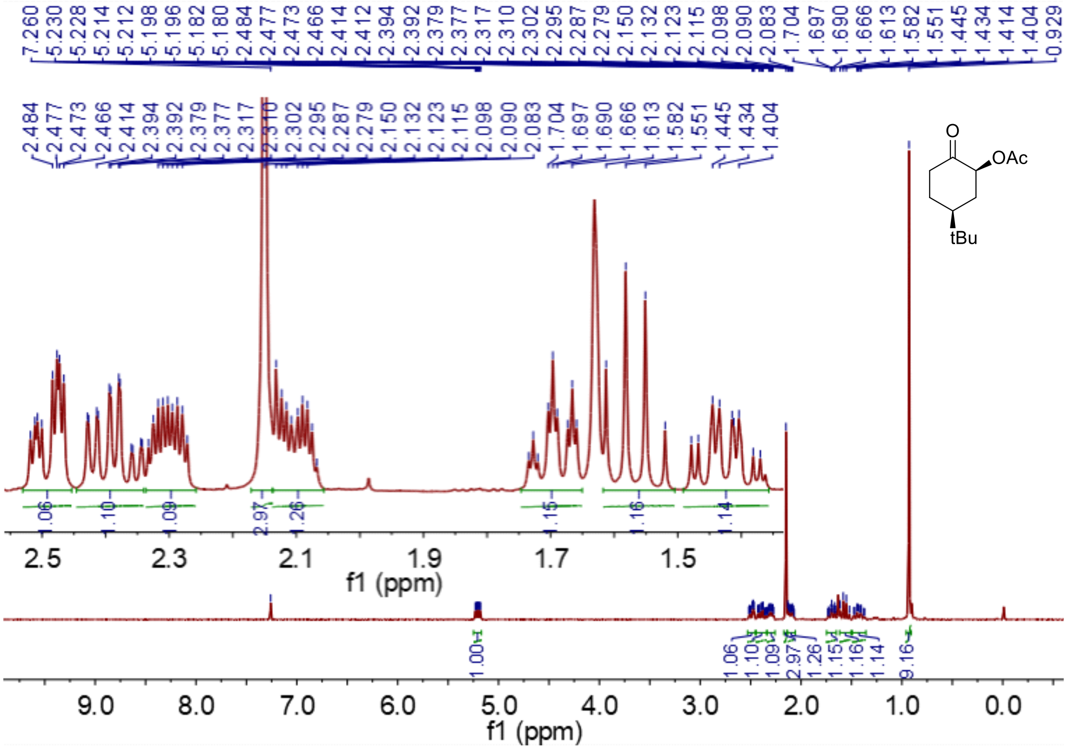

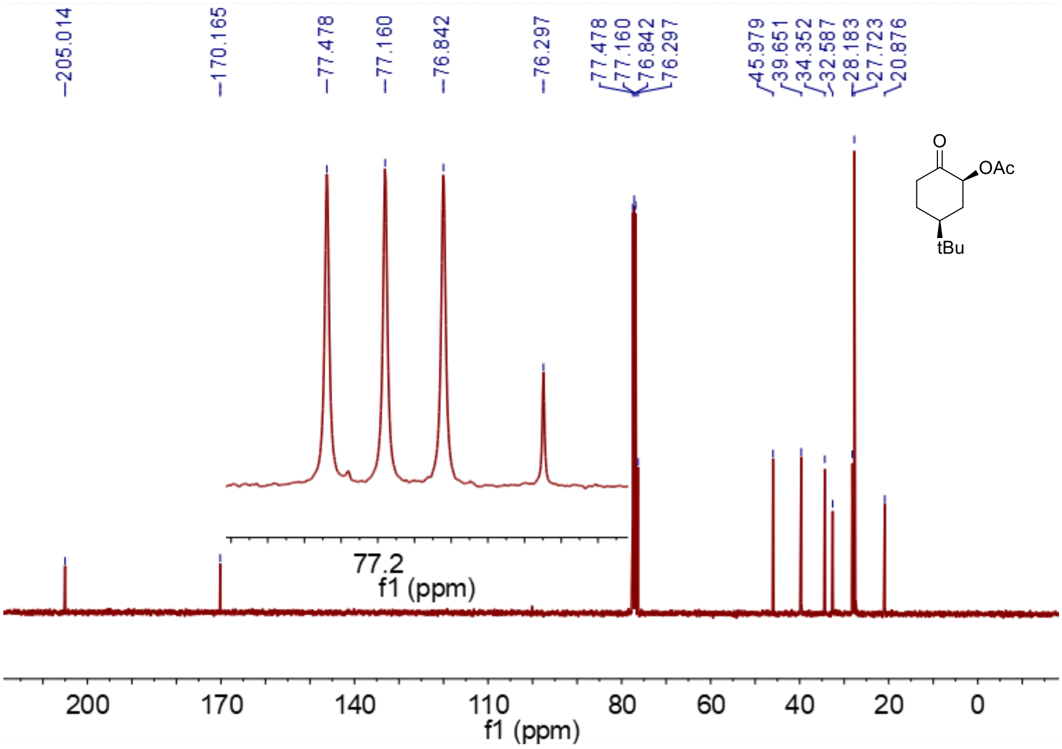

^{1}H and ^{13}C NMR spectra of product *cis*-3

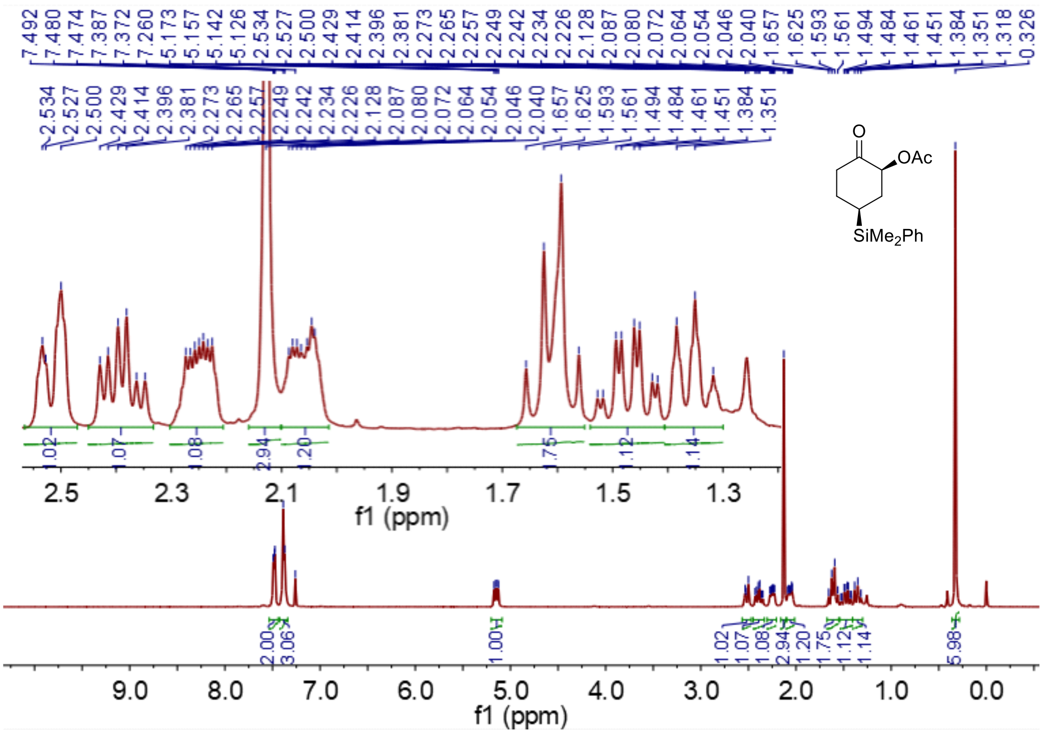

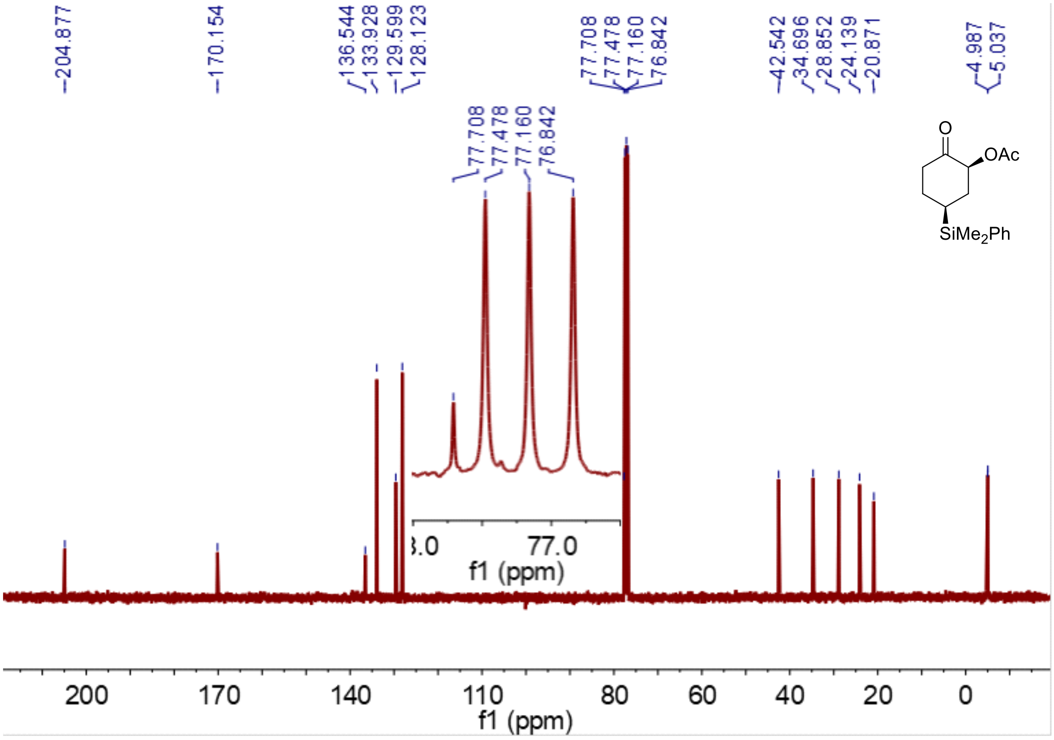

^{1}H and ^{13}C NMR spectra of product *trans*-4

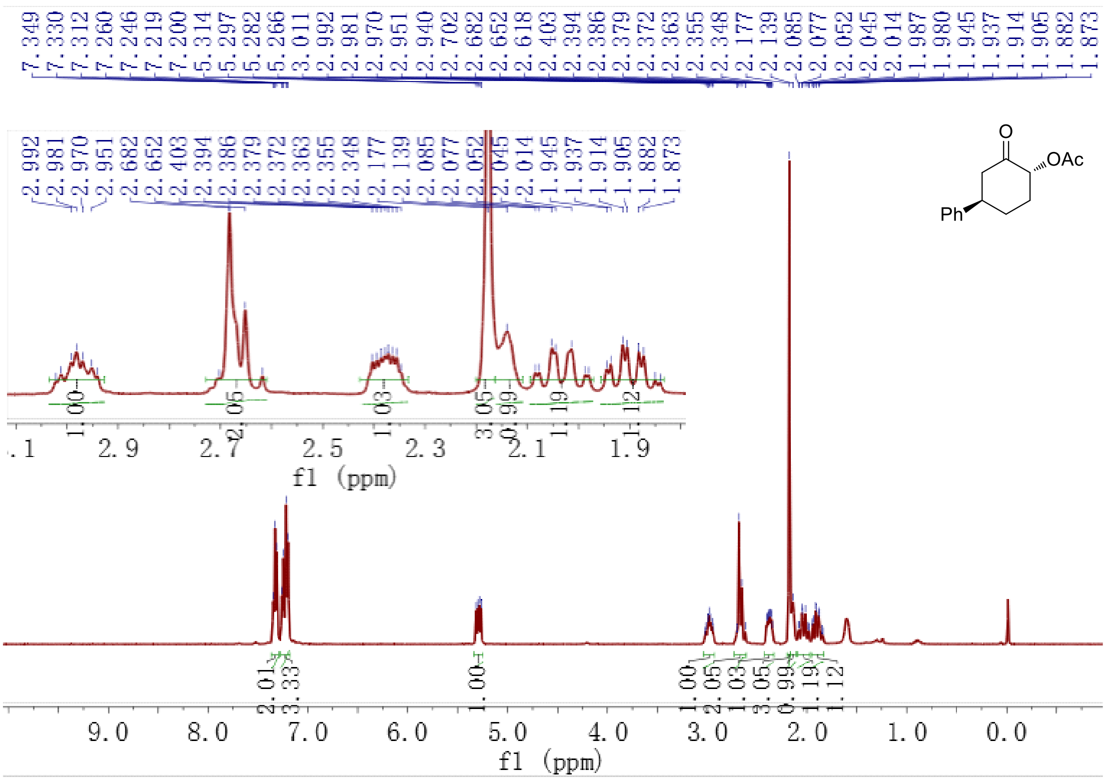

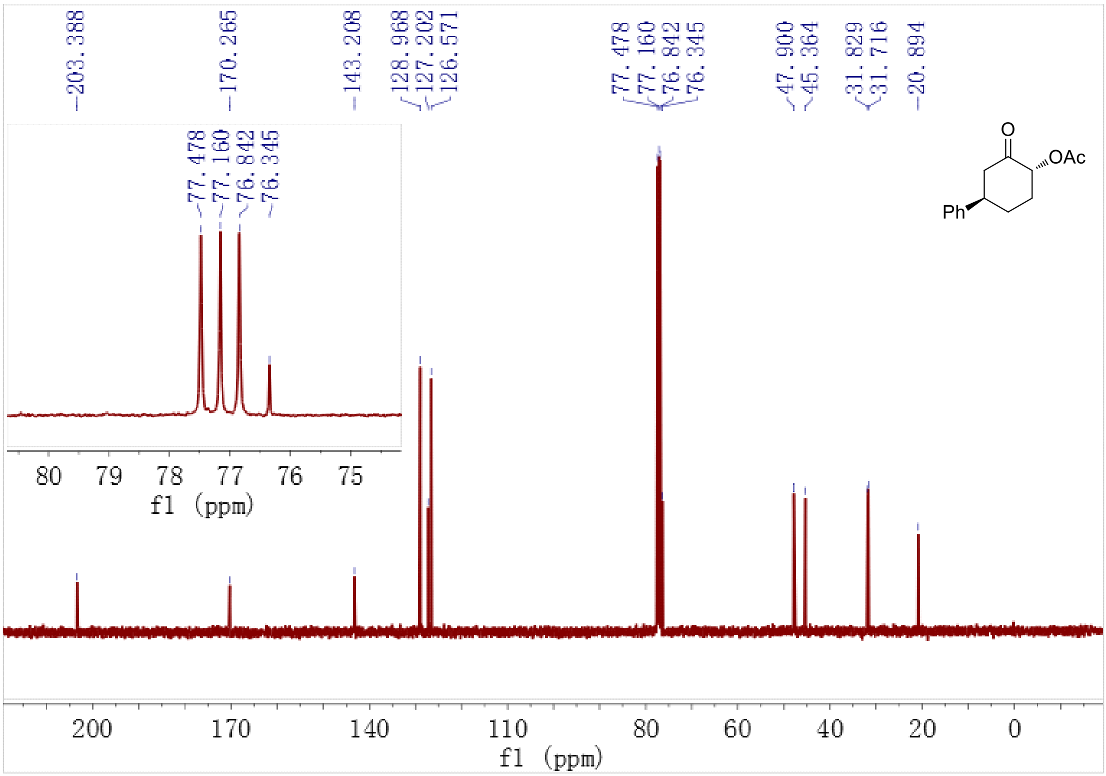

^{1}H and ^{13}C NMR spectra of product *trans*-5

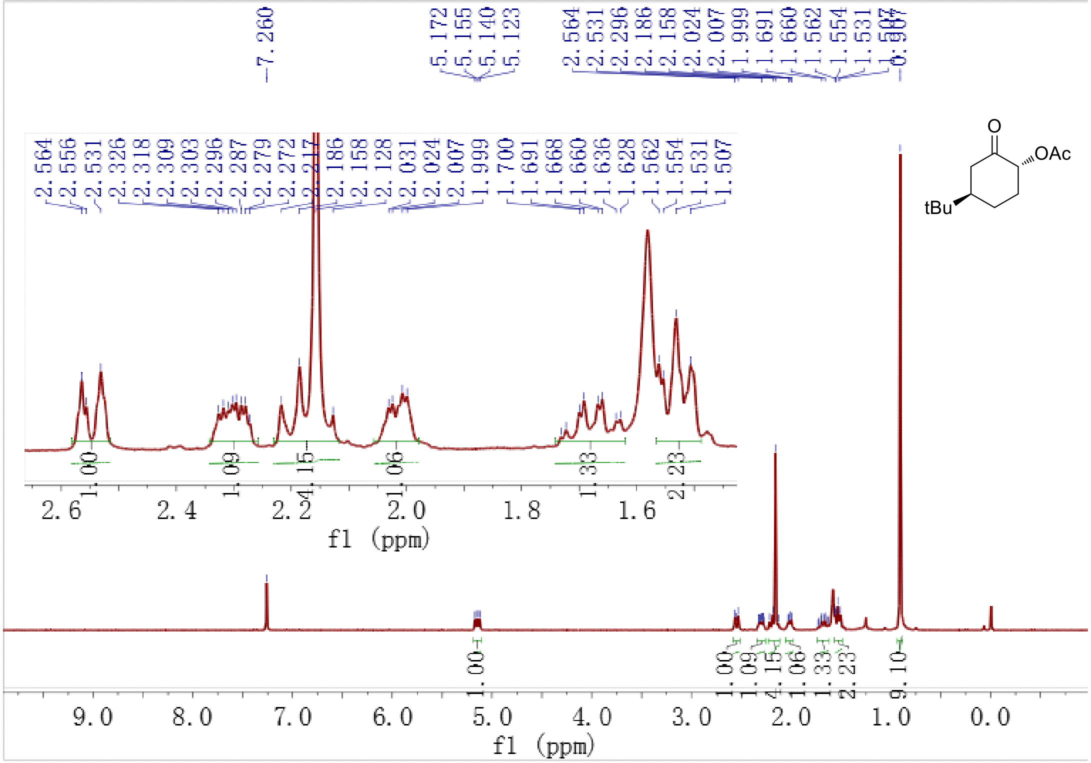

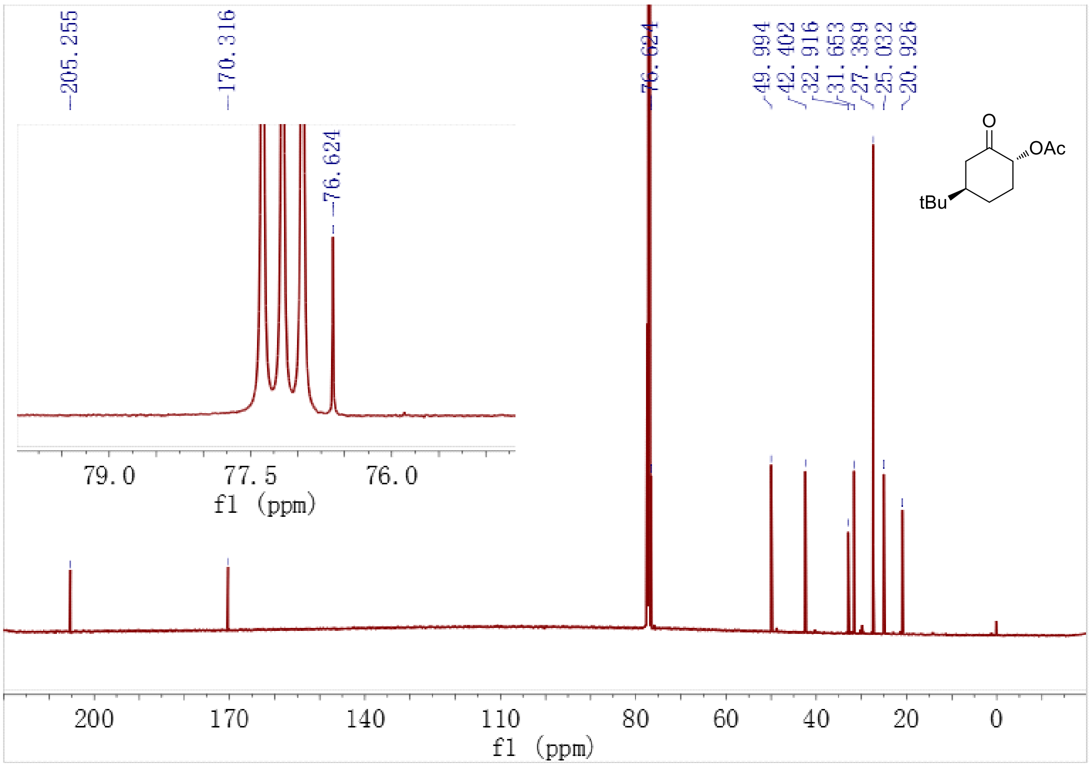

^{1}H and ^{13}C NMR spectra of product *trans*-6

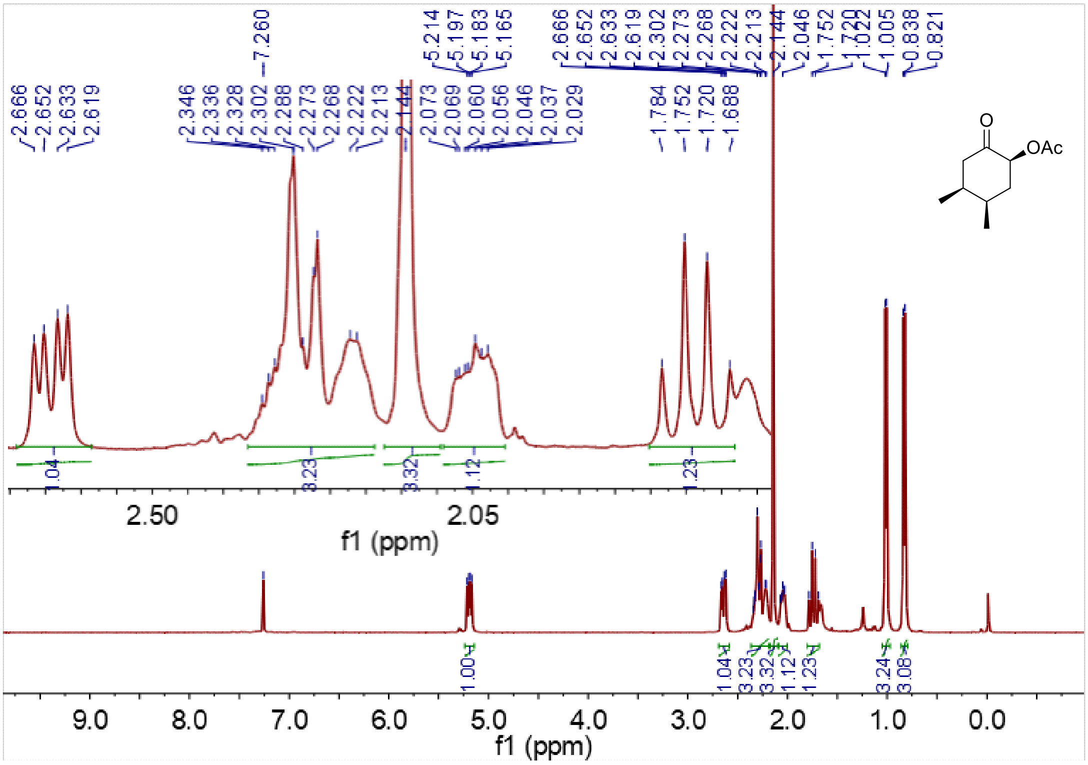

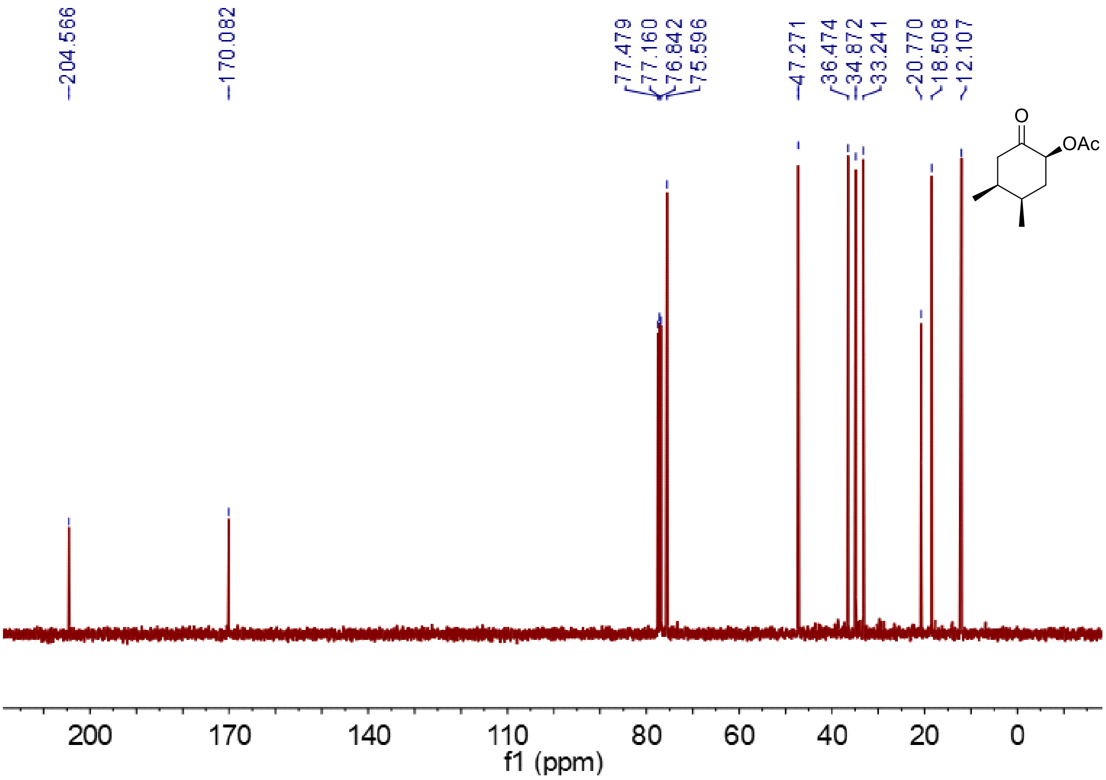

^{1}H and ^{13}C NMR spectra of product 7

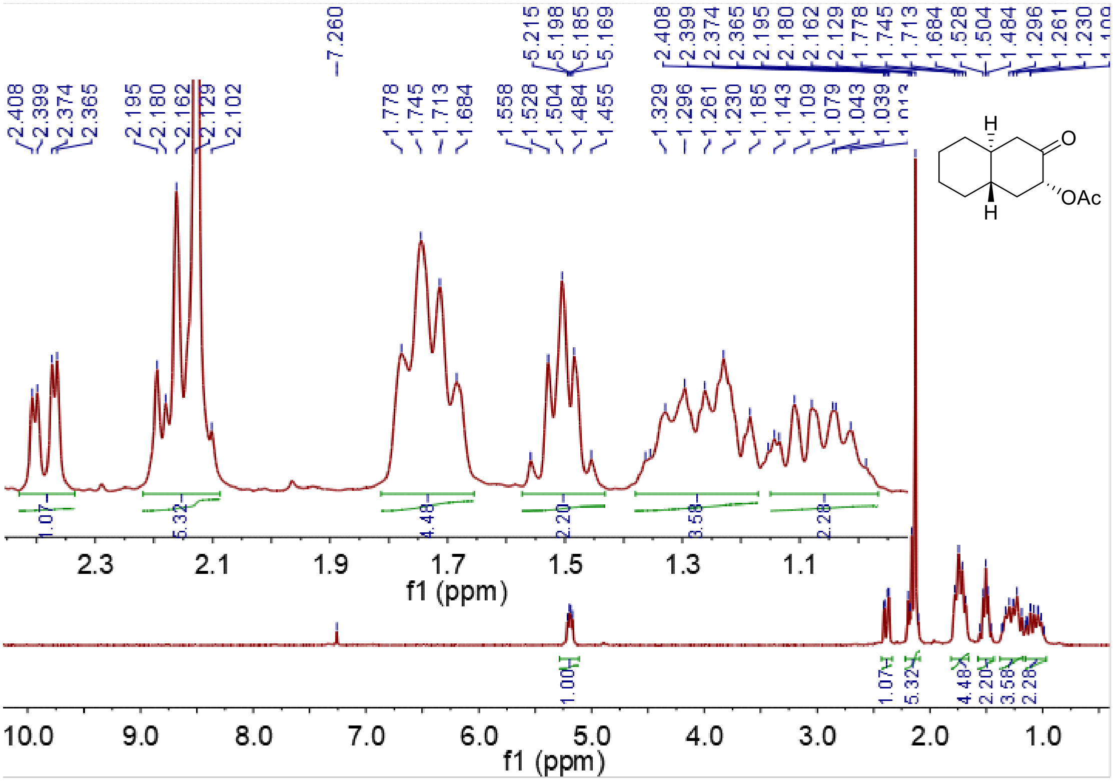

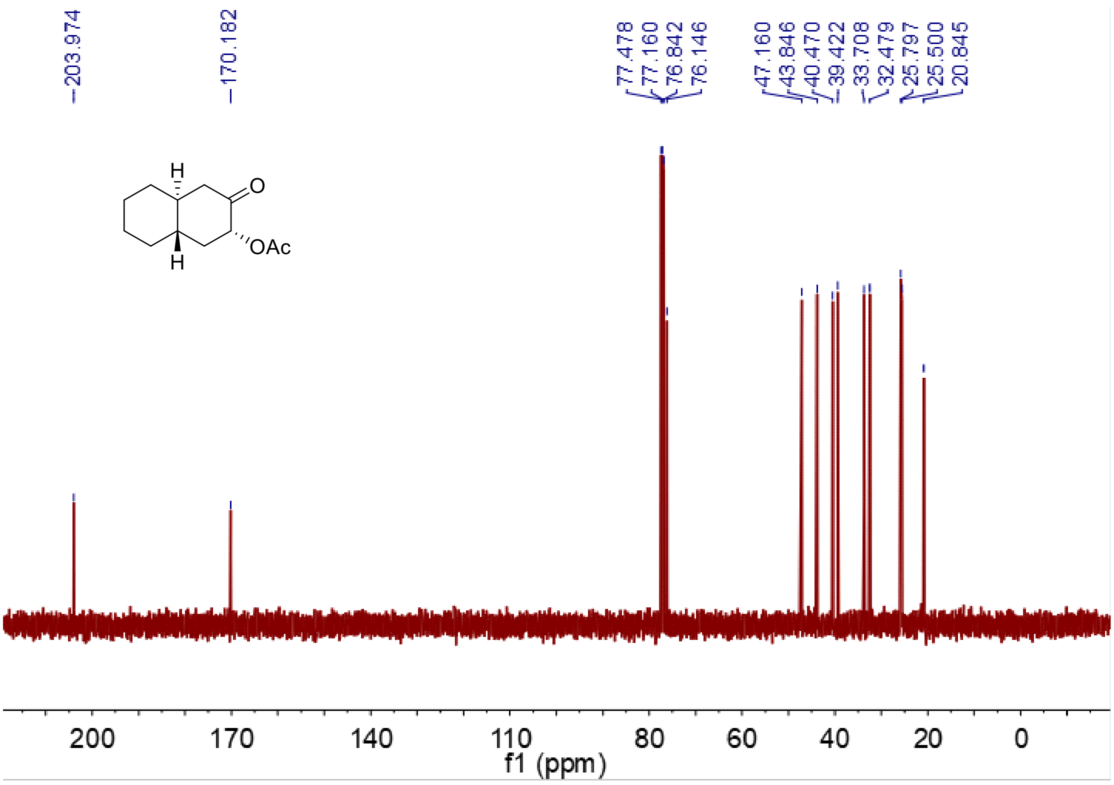

^{1}H and ^{13}C NMR spectra of product *cis*-8

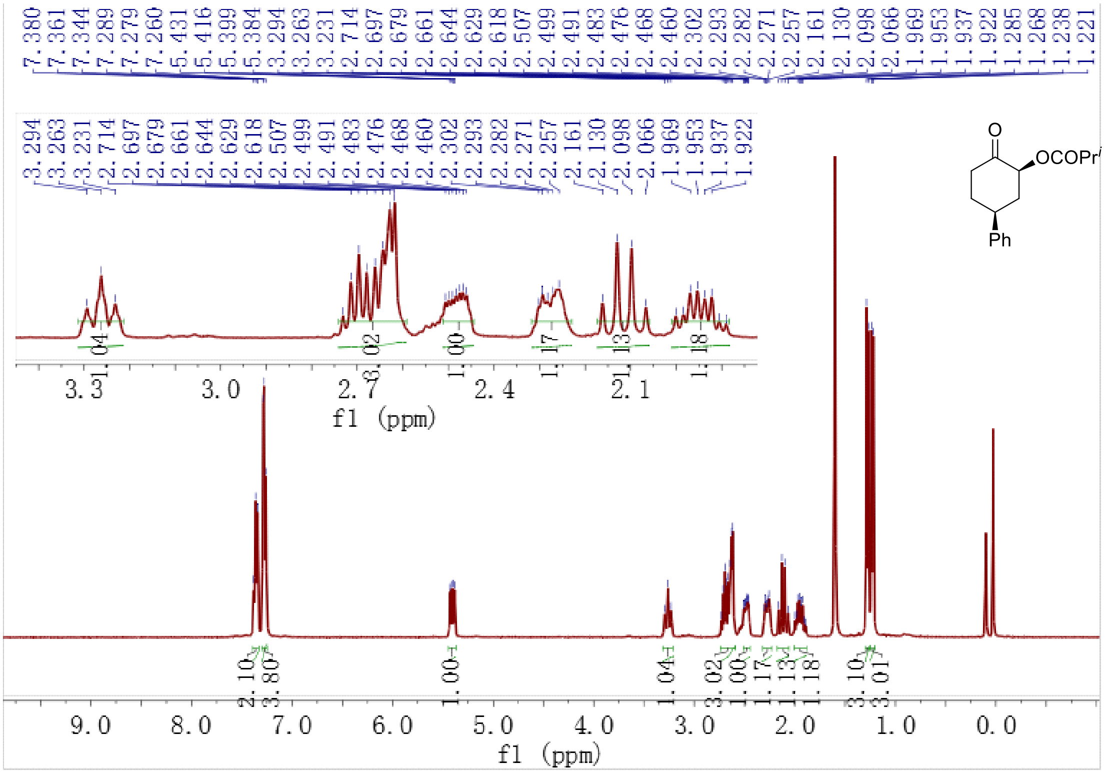

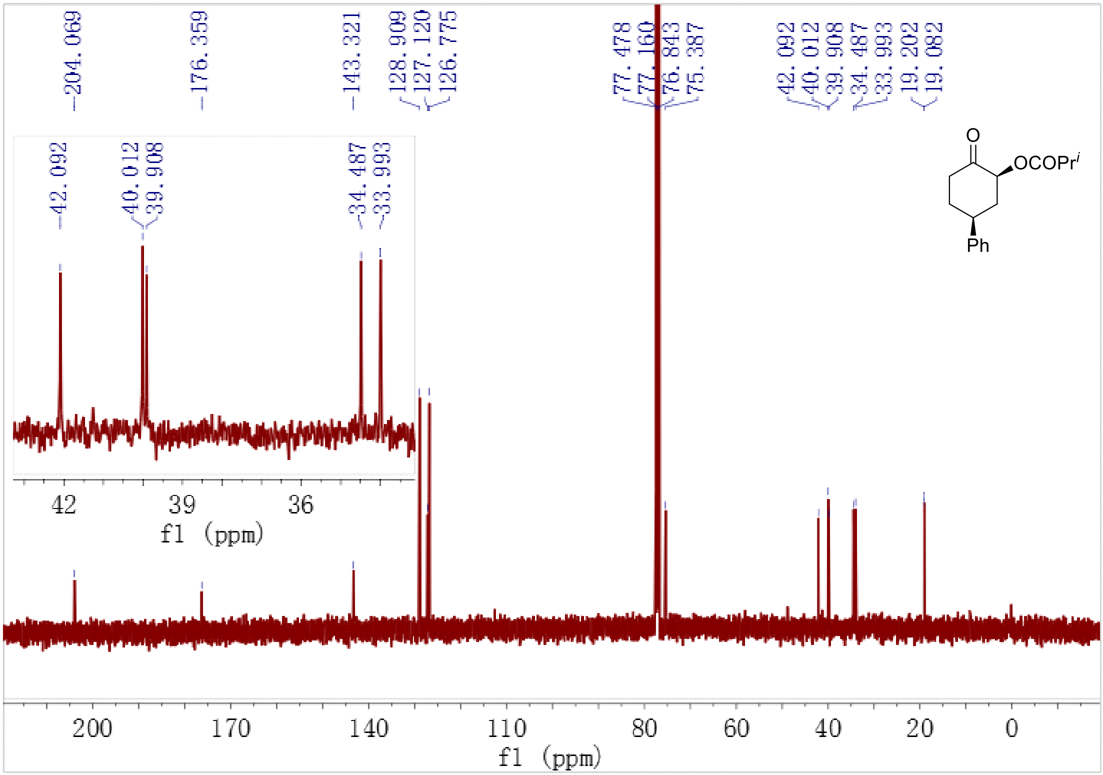

Computational details and references

All the calculations were carried out using the Gaussian 16 program. [1] Geometry optimizations and frequency calculations were carried out with M06-2X functional,[2] at the M06-2X/6-311++G(d, p)&SDD(I)[3]/SMD(acetic acid),[4] level of theory. All the optimized structures were further verified to be stationary points as local minima or transition states via vibrational frequency analyses at the same level of theory. All transition states were confirmed to connect correct reactants and products by intrinsic reaction coordinate (IRC) calculations.

1. M. J. Frisch, G. W. Trucks, H. B. Schlegel, G. E. Scuseria, M. A. Robb, J. R. Cheeseman, G. Scalmani, V. Barone, G. A. Petersson, H. Nakatsuji, X. Li, M. Caricato, A. V. Marenich, J. Bloino, B. G. Janesko, R. Gomperts, B. Mennucci, H. P. Hratchian, J. V. Ortiz, A. F. Izmaylov, J. L. Sonnenberg, D. Williams-Young, F. Ding, F. Lipparini, F. Egidi, J. Goings, B. Peng, A. Petrone, T. Henderson, D. Ranasinghe, V. G. Zakrzewski, J. Gao, N. Rega, G. Zheng, W. Liang, M. Hada, M. Ehara, K. Toyota, R. Fukuda, J. Hasegawa, M. Ishida, T. Nakajima, Y. Honda, O. Kitao, H. Nakai, T. Vreven, K. Throssell, J. A. Montgomery, Jr., J. E. Peralta, F. Ogliaro, M. J. Bearpark, J. J. Heyd, E. N. Brothers, K. N. Kudin, V. N. Staroverov, T. A. Keith, R. Kobayashi, J. Normand, K. Raghavachari, A. P. Rendell, J. C. Burant, S. S. Iyengar, J. Tomasi, M. Cossi, J. M. Millam, M. Klene, C. Adamo, R. Cammi, J. W. Ochterski, R. L. Mar-tin, K. Morokuma, O. Farkas, J. B. Foresman, D. J. Fox, Gaussian 16 Revision A.03, *Gaussian, Inc.*, Wallingford CT, 2016.
2. (a) M. Walker, A. J. A. Harvey, A. Sen, C. E. H. Dessent, *J. Phys. Chem. A,* 2013, **117**, 12590-12600. (b) Y. Zhao, D. G. Truhlar, *Theor. Chem. Account,* 2008, **120**, 215-241.
3. D. Andrae, U. Häuβermann, M. Dolg, H. Stoll, H. Preuβ, *Theor. Chim. Acta*, 1991, **78**, 247-266.
4. A. V. Marenich, C. J. Cramer, D. G. Truhlar, *J. Phys. Chem. B*, 2009, **113**, 6378-6396.

Cartesian coordinates the optimized structures

$I_{transoidal}$

6 0.914999 -0.941535 -0.324291
6 1.625449 -1.569775 0.617890
6 3.104660 -1.360768 0.803045
6 3.591853 -0.075248 0.118351
6 3.035869 -0.018085 -1.311255
6 1.509860 0.047346 -1.291174
1 3.443516 0.846060 -1.842083
1 3.352214 -0.914645 -1.855858
1 1.180741 1.060427 -1.017709
1 1.099144 -0.154613 -2.285537
1 3.659509 -2.221255 0.406632
1 1.113050 -2.276064 1.265336
8 -0.415278 -1.204050 -0.486517
53 -1.746310 0.435649 -0.186248
6 -3.196018 -1.091254 0.053630
6 -4.303736 -1.088851 -0.778089
6 -2.980094 -2.051990 1.028006
6 -5.242708 -2.103549 -0.615857
1 -4.439043 -0.319000 -1.527490
6 -3.927498 -3.062180 1.167792
1 -2.096998 -2.025123 1.654532
6 -5.053837 -3.085860 0.351256
1 -6.118747 -2.123333 -1.253532
1 -3.781631 -3.826367 1.922279
1 -5.788471 -3.873984 0.469320
8 -3.541619 1.707202 0.203307
6 -3.261577 2.976246 0.248070
8 -2.122524 3.409628 0.093230
6 -4.448645 3.867689 0.495953
1 -5.164925 3.744229 -0.318717
1 -4.942454 3.565588 1.421000
1 -4.130945 4.906513 0.559150
6 5.098202 0.047381 0.156450
6 5.710410 1.079957 0.868786
6 5.913167 -0.865264 -0.521590
6 7.097583 1.202920 0.904709
1 5.092715 1.797340 1.400577
6 7.298340 -0.745743 -0.489499
1 5.461137 -1.677872 -1.082140
6 7.896436 0.289990 0.225085
1 7.553360 2.013387 1.463176
1 7.913222 -1.462036 -1.023508
1 8.976341 0.383615 0.249530
1 3.172796 0.774826 0.670489
1 3.338944 -1.317887 1.871947

$I_{cisoidal}$

6 0.673002 -1.035942 -0.903128
6 0.899588 -0.654081 0.357662
6 2.280126 -0.341644 0.878003
6 3.290322 -0.119893 -0.256286
6 3.149426 -1.246839 -1.286348
6 1.763921 -1.212559 -1.926334
1 3.919458 -1.156796 -2.057403

1 3.297693 -2.211263 -0.788162
1 1.689632 -0.392129 -2.650849
1 1.567527 -2.135479 -2.479633
1 2.631746 -1.153826 1.527450
1 0.075349 -0.553244 1.058084
8 -0.543844 -1.294880 -1.462845
53 -2.359908 -0.996325 -0.475027
6 -1.900069 1.091386 -0.491312
6 -2.124861 1.839129 0.651354
6 -1.356920 1.615973 -1.651325
6 -1.796807 3.192261 0.616194
1 -2.531292 1.390606 1.549731
6 -1.033815 2.970517 -1.662202
1 -1.181739 0.994148 -2.520721
6 -1.254515 3.755105 -0.534581
1 -1.960620 3.798476 1.499579
1 -0.612518 3.407162 -2.560248
1 -1.000102 4.808619 -0.551917
8 -4.322778 -0.354402 0.322703
6 -4.553816 -0.369088 1.608643
8 -3.695560 -0.587388 2.454106
6 -5.989887 -0.077347 1.958704
1 -6.270511 0.893541 1.545728
1 -6.631143 -0.831139 1.497781
1 -6.125206 -0.079468 3.038318
6 4.702284 0.014257 0.266629
6 5.400594 1.214743 0.129822
6 5.343226 -1.057860 0.897335
6 6.702835 1.346871 0.607171
1 4.918265 2.056691 -0.357535
6 6.643445 -0.930872 1.374360
1 4.822299 -2.002861 1.016867
6 7.329112 0.273368 1.231052
1 7.226468 2.289439 0.489929
1 7.123787 -1.773666 1.859675
1 8.342421 0.371788 1.603975
1 3.027215 0.820145 -0.756497
1 2.240723 0.552919 1.508082

I_{vert}

6 -0.758228 0.106704 -1.369968
6 -1.566318 1.109976 -1.724490
6 -2.986148 1.228815 -1.240645
6 -3.243454 0.350671 -0.007607
6 -2.690118 -1.057851 -0.263549
6 -1.174605 -1.016448 -0.458944
1 -2.943388 -1.721558 0.567241
1 -3.162575 -1.468322 -1.163095
1 -0.669021 -0.900545 0.510000
1 -0.810625 -1.958306 -0.882187
1 -3.680238 0.950922 -2.044214
1 -1.180189 1.869828 -2.398420
8 0.519762 0.032342 -1.861452
53 2.088464 0.630790 -0.585839
6 2.236316 -1.396231 0.048474
6 2.354580 -1.679733 1.398993
6 2.168881 -2.374378 -0.929249
6 2.399123 -3.017862 1.781304

1 2.413575 -0.888341 2.135473
6 2.215495 -3.705957 -0.524910
1 2.066275 -2.114194 -1.975523
6 2.328886 -4.025693 0.824186
1 2.485909 -3.265641 2.832864
1 2.164595 -4.489561 -1.272042
1 2.362681 -5.064547 1.131751
8 3.815923 0.824097 0.823075
6 4.240887 2.048631 0.927763
8 3.724315 2.984593 0.322886
6 5.415663 2.223030 1.852950
1 5.129143 1.913171 2.859870
1 6.233046 1.577988 1.526255
1 5.737232 3.262552 1.861110
6 -4.704550 0.330027 0.380246
6 -5.119949 0.818882 1.619914
6 -5.672311 -0.188593 -0.486806
6 -6.463284 0.792612 1.988165
1 -4.382261 1.224723 2.305723
6 -7.014379 -0.216907 -0.123373
1 -5.374437 -0.575392 -1.456409
6 -7.415397 0.274375 1.117157
1 -6.765044 1.176950 2.956472
1 -7.749414 -0.623259 -0.809809
1 -8.461823 0.253261 1.400659
1 -2.681802 0.781683 0.830260
1 -3.206767 2.274022 -1.000231

$II_{cisoidal}$

6 0.485609 -2.622019 -0.726638
6 0.357328 -1.121050 -0.846876
6 1.446972 -0.387911 -0.071644
6 2.833244 -0.889276 -0.522679
6 2.944636 -2.401952 -0.297622
6 1.849471 -3.164535 -1.058218
1 1.367938 0.687832 -0.243377
1 2.928213 -0.696175 -1.597942
1 3.923275 -2.758291 -0.626507
1 2.862614 -2.620029 0.772369
1 1.996253 -3.030533 -2.136931
1 1.861537 -4.232140 -0.836916
1 1.324129 -0.569906 1.001339
8 -0.446227 -3.311803 -0.375727
6 3.930988 -0.121363 0.180628
6 4.810369 0.680339 -0.547648
6 4.080756 -0.188083 1.569196
6 5.817660 1.399742 0.090903
1 4.705602 0.741964 -1.626549
6 5.085333 0.529189 2.209708
1 3.408086 -0.803076 2.159265
6 5.957896 1.326289 1.472345
1 6.491010 2.017139 -0.493542
1 5.187896 0.466557 3.287551
1 6.741387 1.883979 1.972942
53 -1.667770 -0.618010 -0.173967
6 -1.249715 1.434955 -0.525122
6 -1.107555 1.868315 -1.834051
6 -1.110601 2.277978 0.566281

6 -0.793403 3.207772 -2.052185
1 -1.242383 1.188656 -2.667056
6 -0.801485 3.613991 0.327488
1 -1.244160 1.908274 1.575240
6 -0.639415 4.075034 -0.975643
1 -0.679713 3.569555 -3.067476
1 -0.689236 4.292213 1.165211
1 -0.399107 5.116856 -1.153687
8 -4.571669 -1.437777 0.930592
6 -4.712710 -0.200005 1.040681
8 -3.859748 0.653996 0.652157
6 -5.968179 0.326352 1.714886
1 -6.825556 -0.298095 1.461520
1 -5.821287 0.271960 2.797561
1 -6.158552 1.364179 1.443443
1 0.371808 -0.844255 -1.905113

II_{vert}

6 -0.137413 -2.257407 0.219217
6 -0.373306 -1.241553 -0.872289
6 -1.820472 -0.782862 -1.000561
6 -2.473662 -0.467753 0.353545
6 -2.337037 -1.675213 1.290580
6 -0.866141 -2.045416 1.519598
1 -1.889995 0.074785 -1.675660
1 -1.941835 0.376510 0.808913
1 -2.809336 -1.458167 2.251160
1 -2.862877 -2.532889 0.857529
1 -0.361491 -1.223724 2.044760
1 -0.756192 -2.947036 2.123111
1 -2.367939 -1.602966 -1.481304
1 0.049836 -1.601437 -1.808383
8 0.617075 -3.187884 0.029243
6 -3.916719 -0.050999 0.169151
6 -4.331701 1.238535 0.503267
6 -4.861972 -0.945192 -0.343153
6 -5.657707 1.628982 0.332387
1 -3.610271 1.945492 0.901810
6 -6.186584 -0.558607 -0.514783
1 -4.564295 -1.954010 -0.612266
6 -6.589544 0.731308 -0.177048
1 -5.960147 2.635654 0.599164
1 -6.906234 -1.265411 -0.913411
1 -7.622314 1.032841 -0.310437
53 0.905841 0.519429 -0.417709
6 2.607866 -0.722510 -0.134579
6 3.008156 -1.004135 1.161931
6 3.243841 -1.234468 -1.253928
6 4.096621 -1.853987 1.337232
1 2.497829 -0.572327 2.013989
6 4.328911 -2.083394 -1.054968
1 2.911009 -0.985021 -2.253841
6 4.750582 -2.392832 0.234056
1 4.428459 -2.091387 2.341164
1 4.841767 -2.499702 -1.914113
1 5.594989 -3.056434 0.379688
8 1.056657 3.669452 -0.000778
6 2.264043 3.421026 0.208782

8 2.776540 2.261854 0.138120
6 3.179316 4.572042 0.587131
1 3.064144 5.380955 -0.136845
1 2.870107 4.956574 1.562396
1 4.220582 4.257204 0.635050

axial-II_{vert}

6 0.130690 -2.738724 0.231020
6 -0.123054 -1.878260 -0.979219
6 -1.501349 -2.078503 -1.596535
6 -2.655950 -2.052145 -0.578103
6 -2.363473 -3.082885 0.519728
6 -1.004177 -2.882889 1.209580
1 -1.650910 -1.367008 -2.409549
1 -3.155819 -3.087824 1.271082
1 -2.372186 -4.073456 0.055479
1 -1.005099 -1.963982 1.808404
1 -0.770077 -3.710178 1.880863
1 -1.477923 -3.072965 -2.058454
1 0.680793 -2.012116 -1.701534
8 1.212444 -3.264971 0.393066
53 0.232087 0.241716 -0.364566
6 2.283431 -0.204991 -0.032835
6 2.684931 -0.552466 1.247312
6 3.160246 -0.129326 -1.103539
6 4.029451 -0.847586 1.454949
1 1.975510 -0.595199 2.064759
6 4.500541 -0.428490 -0.875118
1 2.817709 0.161318 -2.089125
6 4.931781 -0.787134 0.398358
1 4.365117 -1.125518 2.447133
1 5.205170 -0.374083 -1.696822
1 5.977210 -1.017027 0.568937
8 -0.573721 3.395526 -0.517207
6 0.530160 3.498963 0.056868
8 1.286634 2.518446 0.337771
6 1.003207 4.885243 0.459857
1 0.977617 5.543392 -0.411053
1 0.309238 5.291869 1.199754
1 2.008485 4.860527 0.877604
6 -2.951506 -0.641427 -0.080249
6 -3.073443 -0.321850 1.274019
6 -3.123861 0.391444 -1.013720
6 -3.297620 0.991032 1.684329
1 -2.988741 -1.091055 2.032274
6 -3.344569 1.701553 -0.607082
1 -3.082357 0.174753 -2.076216
6 -3.414276 2.010416 0.747838
1 -3.370510 1.214126 2.743119
1 -3.445023 2.485603 -1.348884
1 -3.561070 3.035738 1.066712
1 -3.547552 -2.391695 -1.116247

cis-TS

6 -0.87228600 1.27874100 1.02229400
6 -0.31049200 0.90555000 -0.33768300

6 1.16547600 0.81234900 -0.59943400
6 2.02941400 0.73282100 0.67136900
6 1.53299800 1.73598700 1.71319600
6 0.11328500 1.39033100 2.15598000
1 1.38108200 -0.02667700 -1.26418700
1 1.91404900 -0.27596900 1.08569000
1 2.19129500 1.72421000 2.58586700
1 1.54214200 2.74853500 1.30172400
1 0.10806600 0.41065200 2.65283400
1 -0.28947000 2.11475600 2.86628000
1 1.41638600 1.72277600 -1.15293100
1 -0.99943000 1.01338800 -1.16697600
8 -2.06323800 1.44094700 1.17037800
6 3.48870900 0.90654200 0.29088800
6 4.15832500 -0.14913000 -0.33884400
6 4.18554300 2.09602400 0.51207100
6 5.48631900 -0.02091000 -0.72937200
1 3.62146400 -1.07633100 -0.51639500
6 5.51694200 2.22631000 0.11949200
1 3.69771700 2.93540300 0.99426300
6 6.17318400 1.16951600 -0.50112000
1 5.98709700 -0.85364800 -1.21216000
1 6.04017200 3.15867700 0.30276600
1 7.20944300 1.27081000 -0.80407100
53 -1.0093610 -1.5935210 -0.15419500
6 -3.05610700 -1.01926600 -0.07576300
6 -3.78943900 -1.29882300 1.07074500
6 -3.61797900 -0.34279000 -1.15120600
6 -5.11667800 -0.88335300 1.13964300
1 -3.33315400 -1.81966400 1.90415600
6 -4.94108300 0.07791600 -1.06445200
1 -3.03324800 -0.12005300 -2.03626900
6 -5.68948900 -0.19109000 0.07769900
1 -5.69586700 -1.09446400 2.03155800
1 -5.38152600 0.62326400 -1.89129800
1 -6.71894100 0.14293500 0.14052400
8 0.98747600 -4.48441400 -0.08020700
6 1.89085200 -3.62275500 -0.17403700
8 1.70470800 -2.37753500 -0.28163500
6 3.34450700 -4.08575000 -0.15524100
1 3.41577200 -5.17143700 -0.09451100
1 3.85074500 -3.73539600 -1.05807500
1 3.85412600 -3.63820300 0.70200800
8 -2.11389800 2.99548900 -1.63038900
6 -1.34421200 3.62506500 -0.88594000
8 -0.28440400 3.14402300 -0.35788200
6 -1.66357600 5.06290900 -0.51795600
1 -2.38976700 5.49117000 -1.20789900
1 -2.08788500 5.06111500 0.49056400
1 -0.75459900 5.66553500 -0.49724700

trans-TS

6 -0.83067900 -2.03372300 -0.31307900
6 -1.33656300 -0.71392200 0.23018800
6 -1.08718100 -0.25192500 1.63233100
6 0.06990400 -0.96584700 2.35999500
6 -0.00323000 -2.47481100 2.08486400
6 0.01103600 -2.87424600 0.60492800

1 -0.97795600 0.83533500 1.64719900
1 0.81354000 -2.98623800 2.60154900
1 -0.93390500 -2.83716500 2.52880800
1 1.01065600 -2.81156900 0.16378400
1 -0.31747700 -3.90871400 0.47841600
1 -2.02119800 -0.45994800 2.16190000
1 -2.13255900 -0.25799800 -0.34567300
8 -1.08370400 -2.35507100 -1.45217600
53 0.33715400 0.77722300 -1.14126800
6 -0.89424100 2.41538600 -0.56458400
6 -2.21103400 2.48369300 -1.00966500
6 -0.36828200 3.39501000 0.27185600
6 -3.01376500 3.54405100 -0.59754700
1 -2.61221100 1.72035500 -1.66753200
6 -1.17892600 4.45454000 0.67287800
1 0.66115600 3.33374800 0.60607900
6 -2.49984200 4.52825300 0.24189300
1 -4.04145900 3.59736400 -0.93875800
1 -0.77353000 5.22085900 1.32413200
1 -3.12840300 5.35315300 0.55778400
8 3.57361000 -0.12205100 -2.07822500
6 3.09897500 -1.23725000 -1.76349800
8 1.87116500 -1.49896800 -1.62778800
6 4.06109800 -2.40042200 -1.53978100
1 5.09814200 -2.06528300 -1.51987700
1 3.81530700 -2.91843300 -0.60949300
1 3.93503300 -3.11995700 -2.35356200
6 1.41142800 -0.29427800 2.09723100
6 2.51816900 -0.95014900 1.56040400
6 1.54286600 1.06347500 2.41467300
6 3.69772300 -0.25759700 1.28828000
1 2.47556800 -2.00833700 1.33530700
6 2.71716300 1.75584000 2.15231400
1 0.70680900 1.58917200 2.86819300
6 3.79789300 1.09905600 1.56683400
1 4.53800000 -0.78140200 0.84548700
1 2.79036200 2.80957700 2.40043400
1 4.70957300 1.63885200 1.33753600
1 -0.12815700 -0.83073100 3.42854800
8 -4.11154400 -1.33566600 -0.76472900
6 -3.88073700 -2.18395800 0.11103500
8 -2.85105900 -2.18865500 0.86957500
6 -4.84640800 -3.33593200 0.32357500
1 -4.98197900 -3.52829700 1.38877400
1 -5.80562100 -3.13314400 -0.15100500
1 -4.40878100 -4.23222200 -0.12480600

cis-1

6 1.756240 1.301865 0.241153
6 1.508411 -0.080312 -0.347191
6 0.093043 -0.540100 -0.008368
6 -0.942515 0.480661 -0.504173
6 -0.667465 1.858605 0.115505
6 0.759757 2.341190 -0.192597
1 -0.077773 -1.517690 -0.465409
1 0.872475 2.483623 -1.273799
1 0.988882 3.281977 0.308658
1 0.013800 -0.659379 1.077800

8 2.652507 1.506198 1.027073
8 2.421369 -1.025859 0.195533
6 3.697443 -0.956011 -0.231479
8 4.051677 -0.194366 -1.097229
6 4.566972 -1.932264 0.491322
1 5.576845 -1.892214 0.090566
1 4.574056 -1.679378 1.553758
1 4.153789 -2.937223 0.387432
1 1.637501 -0.016574 -1.432890
1 -0.800894 1.803416 1.201087
1 -1.386519 2.588744 -0.262803
1 -0.833534 0.571960 -1.591590
6 -2.351374 0.003379 -0.221595
6 -3.232822 -0.268843 -1.268256
6 -2.796364 -0.182725 1.090995
6 -4.527850 -0.715208 -1.015126
1 -2.901607 -0.131526 -2.293044
6 -4.088661 -0.627127 1.347465
1 -2.128500 0.018790 1.922758
6 -4.959980 -0.895176 0.294180
1 -5.197501 -0.922375 -1.842622
1 -4.416996 -0.765420 2.371742
1 -5.967460 -1.242118 0.494329

trans-1

6 1.331294 1.209497 -0.642491
6 1.163628 -0.004680 0.258325
6 0.302212 0.344820 1.475980
6 -1.028264 1.014129 1.102572
6 -0.745670 2.232995 0.203732
6 0.035922 1.839956 -1.061478
1 0.147603 -0.558055 2.071126
1 -0.541550 1.107744 -1.638221
1 0.248086 2.703393 -1.692569
1 0.878341 1.041808 2.092117
8 2.424097 1.641145 -0.930338
8 2.431767 -0.436114 0.739661
6 3.249104 -1.036732 -0.148685
8 2.890542 -1.344762 -1.258429
6 4.612283 -1.250187 0.423488
1 5.197329 -1.882381 -0.240269
1 5.094900 -0.274749 0.525126
1 4.538579 -1.694567 1.416701
1 0.706322 -0.793946 -0.345218
1 -1.457445 1.396023 2.035278
1 -0.157837 2.958663 0.774990
1 -1.671628 2.731333 -0.084975
6 -2.072386 0.082086 0.495889
6 -1.874793 -1.289062 0.322782
6 -3.314203 0.614126 0.125798
6 -2.871376 -2.096156 -0.225660
1 -0.945789 -1.757746 0.624537
6 -4.310980 -0.186467 -0.417337
1 -3.512488 1.670008 0.278772
6 -4.090211 -1.549153 -0.604088
1 -2.687654 -3.157210 -0.353992
1 -5.263662 0.253180 -0.691555
1 -4.863735 -2.176690 -1.031957

trans-4

6 1.250345 -0.679591 -0.509310
6 1.827702 0.439456 0.345638
6 1.014598 1.713491 0.132460
6 -0.461928 1.460855 0.434630
6 -1.031615 0.336789 -0.442688
6 -0.206586 -0.958053 -0.258226
1 1.418006 2.496792 0.777457
1 -0.310496 -1.309735 0.774972
1 -0.542900 -1.746463 -0.932738
1 1.139812 2.036911 -0.906265
8 1.911600 -1.242834 -1.350952
8 3.176363 0.701697 -0.021597
6 4.090805 -0.218823 0.340659
8 3.815270 -1.171092 1.028636
6 5.439089 0.100039 -0.217240
1 6.185021 -0.553281 0.229252
1 5.408770 -0.059689 -1.297977
1 5.683489 1.147363 -0.036705
1 1.779306 0.129017 1.394893
1 -0.582546 1.198068 1.491809
1 -1.032203 2.377464 0.265000
6 -2.499974 0.089254 -0.173728
6 -3.446359 0.270152 -1.182733
6 -2.939338 -0.318387 1.089501
6 -4.800641 0.051793 -0.939492
1 -3.120375 0.584711 -2.169302
6 -4.290299 -0.535961 1.336248
1 -2.221133 -0.469759 1.889522
6 -5.226479 -0.351092 0.321296
1 -5.521592 0.196235 -1.736610
1 -4.613939 -0.851661 2.321939
1 -6.279626 -0.522414 0.513677
1 -0.931379 0.644735 -1.489701

cis-4

6 -0.973390 -0.224157 0.988443
6 -1.544933 -0.703189 -0.339001
6 -0.421492 -0.978210 -1.332284
6 0.551162 -1.996317 -0.740625
6 1.199336 -1.480013 0.559764
6 0.100290 -1.106806 1.574309
1 -0.859926 -1.349800 -2.260683
1 -0.402971 -2.031890 1.880962
1 0.502992 -0.630767 2.469552
1 0.093188 -0.038271 -1.558080
8 -1.368291 0.792189 1.512234
8 -2.405502 0.290115 -0.882351
6 -3.615787 0.430849 -0.307747
8 -4.026720 -0.324724 0.538597
6 -4.344128 1.615747 -0.853030
1 -5.374802 1.605864 -0.506549
1 -3.843020 2.519022 -0.496116
1 -4.302294 1.614467 -1.942860
1 -2.108735 -1.623103 -0.147617

1 0.017764 -2.928705 -0.525416
1 1.772774 -2.303410 0.995397
1 1.326489 -2.231188 -1.472654
6 2.183857 -0.358738 0.266465
6 1.976117 0.969385 0.640055
6 3.355013 -0.672663 -0.434300
6 2.904630 1.957213 0.310600
1 1.093937 1.257582 1.199479
6 4.282453 0.307533 -0.761195
1 3.542654 -1.703451 -0.721412
6 4.057616 1.632692 -0.391954
1 2.719985 2.983125 0.609653
1 5.182990 0.038563 -1.302364
1 4.778604 2.401371 -0.646134

cis-6

6 0.125279 1.067113 -0.145000
6 0.445424 -0.403788 -0.371577
6 -0.714769 -1.263406 0.122620
6 -2.040792 -0.829565 -0.514527
6 -2.329377 0.664784 -0.259252
6 -1.155424 1.516720 -0.789459
1 -0.501541 -2.308332 -0.117404
1 -1.058266 1.370765 -1.871140
1 -1.303617 2.578514 -0.587201
1 -0.771484 -1.183162 1.213385
8 0.826094 1.771210 0.546292
8 1.617522 -0.774006 0.347037
6 2.789383 -0.301638 -0.120565
8 2.878176 0.301463 -1.161851
6 3.919509 -0.629028 0.800197
1 4.860327 -0.326809 0.346464
1 3.770718 -0.094395 1.740971
1 3.924487 -1.698152 1.018067
1 0.601606 -0.558388 -1.444075
6 -3.175783 -1.735172 -0.043880
1 -3.239248 -1.760122 1.047177
1 -4.139470 -1.393278 -0.432047
1 -3.019683 -2.760075 -0.389869
6 -2.610873 0.995052 1.207285
1 -2.800362 2.065343 1.322766
1 -3.490773 0.462218 1.573497
1 -1.767154 0.743791 1.857869
1 -1.940877 -0.948588 -1.601385
1 -3.214461 0.934904 -0.843957

tans-6

6 -0.287854 1.419668 -0.292569
6 0.515307 0.141690 -0.158359
6 -0.124061 -0.765647 0.897573
6 -1.591417 -1.074894 0.574733
6 -2.384775 0.241573 0.462127
6 -1.749481 1.196566 -0.569473
1 0.459512 -1.687278 0.963230
1 -1.838495 0.761951 -1.572984
1 -2.253591 2.164627 -0.574791

1 -0.053363 -0.251521 1.862348
8 0.210786 2.515036 -0.160595
8 1.847640 0.489798 0.212041
6 2.821790 -0.380754 -0.096457
8 2.611283 -1.424278 -0.667506
6 4.160206 0.117478 0.344102
1 4.912713 -0.647158 0.166096
1 4.405845 1.021998 -0.216552
1 4.124522 0.380302 1.402531
1 0.528451 -0.353727 -1.134783
6 -3.858872 0.024187 0.137192
1 -4.313410 -0.680121 0.839843
1 -4.411422 0.965197 0.198898
1 -3.987572 -0.372737 -0.873676
6 -1.725037 -1.982603 -0.651148
1 -1.062979 -2.846949 -0.551600
1 -2.744519 -2.359683 -0.755607
1 -1.462448 -1.476070 -1.584123
1 -1.997562 -1.621057 1.433319
1 -2.318193 0.734197 1.440011
